# The interplay between somatic and dendritic inhibition promotes the emergence and stabilization of place fields

**DOI:** 10.1371/journal.pcbi.1007955

**Published:** 2020-07-10

**Authors:** Victor Pedrosa, Claudia Clopath

**Affiliations:** 1 Department of Bioengineering, Imperial College London, London, United Kingdom; 2 CAPES Foundation, Ministry of Education of Brazil, Brasilia - DF, Brazil; University College London, UNITED KINGDOM

## Abstract

During the exploration of novel environments, place fields are rapidly formed in hippocampal CA1 neurons. Place cell firing rate increases in early stages of exploration of novel environments but returns to baseline levels in familiar environments. Although similar in amplitude and width, place fields in familiar environments are more stable than in novel environments. We propose a computational model of the hippocampal CA1 network, which describes the formation, dynamics and stabilization of place fields. We show that although somatic disinhibition is sufficient to form place fields, dendritic inhibition along with synaptic plasticity is necessary for place field stabilization. Our model suggests that place cell stability can be attributed to strong excitatory synaptic weights and strong dendritic inhibition. We show that the interplay between somatic and dendritic inhibition balances the increased excitatory weights, such that place cells return to their baseline firing rate after exploration. Our model suggests that different types of interneurons are essential to unravel the mechanisms underlying place field plasticity. Finally, we predict that artificially induced dendritic events can shift place fields even after place field stabilization.

## Author summary

Hippocampal pyramidal neurons are thought to encode spatial information. A subset of these cells, named place cells, are active only when the animal traverses a specific region within the environment. Although vastly studied experimentally, the development and stabilization of place fields are not fully understood. Here, we propose a mechanistic model of place cell formation in the hippocampal CA1 region. Using our model, we reproduce place field dynamics observed experimentally and provide a mechanistic explanation for the stabilization of place fields. Finally, our model provides specific predictions on protocols to shift place field location.

## Introduction

The hippocampus encodes spatial information through a subset of pyramidal cells—the place cells—that fires action potentials when the animal is in a specific location within the environment—the place fields [1, 2, 3, 4]. These neurons are thought to encode and store new memories by taking part in activity-dependent synaptic plasticity [5, 6, 7, 8, 9]. How these place fields are formed is not clear and recent experimental data, while unravelling specific parts of the mechanisms underlying place cell dynamics, have also opened up some puzzling questions, especially when put together [9, 10, 11, 12, 13, 14, 15, 16]. Although here we focus on the role of hippocampal cells in spatial memory development, the hippocampus is also associated with other types of memories [17, 18], and the principles governing place field dynamics are likely to be common across several types of hippocampal memory formation.

Subthreshold responses of silent cells, when recorded at the soma, are not place-tuned [13]. If a spatially uniform current is applied to a silent cell, however, this cell starts to produce place-tuned activity [12]. This transition from silent to place cell is abrupt and once the silent cell is turned into a place cell, the amplitude of the place field is fairly independent of the amplitude of the applied current [12]. Furthermore, once the external, spatially uniform current is removed, the cell returns to its silent, untuned state [12]. These results suggest that silent cells receive place-tuned inputs even though there is no signature of those inputs at the soma. Therefore, inputs from dendrites are thought to be nonlinearly propagated to the soma with the somatic depolarization acting as a gate for this propagation [12]. The functional consequences of this gating for the hippocampal network have not been fully explored. For instance, it is not clear which elements of the network are responsible for modulating this dendrite-to-soma propagation.

There is increasing evidence suggesting that place fields are not formed from homogeneously distributed place-tuned inputs [10, 11, 13, 14, 19, 20, 21, 22, 23, 24, 25, 26, 27, 28]. Instead, spatial representation might be built from the selection of already strong connections, without the need for synaptic plasticity [12, 23, 24, 25]. For instance, many place fields, although not stable, are present from the animal’s first traversal of a novel environment [11, 13, 14, 26]. Furthermore, additional place cells are formed mainly during the first few laps of exploration [11]. This poses a question for the role of synaptic plasticity in place field development.

During the exploration of a novel linear track, new place fields are formed over several laps [11]. The development of these new place fields has been shown to be preceded by dendritic regenerative events—backpropagating action potentials or dendritically generated spikes [11, 29]—which are promoted by a reduction in dendritic inhibition through the suppression of somatostatin-expressing (SST) interneuron activity [11]. These dendritic events can be associated with a myriad of factors such as dendritic disinhibition [16, 30, 31], back-propagating action potentials [7, 32, 33, 34], NMDA spikes [35, 36, 37], or plateau potentials [38, 39, 40, 41]. More recently, the conjunctive activation of presynaptic inputs and postsynaptic calcium plateau potentials have been applied to artificially induce new place fields [15, 38]. Additionally, place fields have also been induced following juxtacellular stimulation of CA1 silent cells [42]. Although dendritic disinhibition has been implicated in place field development [11], it is still not clear which role the different types of interneuron play in place field formation and stabilization.

CA1 pyramidal cell depolarization is initially low but rapidly increases during exploration of novel environments [10], which might be linked to a quick increase in place cell firing rate in early stages of exploration [10, 14]. Surprisingly, in familiar environments, subthreshold ramp of depolarization associated with place field firing returns to a lower level, comparable to the level observed during the initial exploration of novel environments [10]. Remarkably, although the level of CA1 pyramidal cell depolarization is similar in the first stages of exploration of novel environments and in familiar environments, place fields in familiar environments have been shown to be considerably more stable [4, 10, 21, 22], and complex spike-mediated synaptic plasticity has been suggested to be involved in this stabilization [10]. Moreover, the blockage of NMDA receptors in CA1 neurons has been shown to significantly decrease the number of new place fields being formed across the network [11]. These results suggest that synaptic plasticity is not required for the formation of place fields but is involved in the development of new place cells and stabilization of spatial representations. Therefore, these results lead to the question of what role synaptic plasticity plays in place field stabilization.

Several computational models have been proposed to account for place field development [16, 43, 44, 45, 46, 47, 48, 49, 50, 51, 52, 53, 54, 55, 56]. The interaction between excitatory and inhibitory plasticity has been shown to lead to the development of place fields in initially untuned pyramidal cells [43]. Alternatively, attractor network models have been proposed as more abstract models of hippocampal circuit dynamics [50, 51, 52, 53, 54, 55, 56] and some models have even been extended to more than two dimensions [47]. Even though these models account for the origin of spatially tuned inputs onto place cells, they do not take into account individual interneuron types and their modulation during exploration of novel environments.

All these questions call for a simplified computational model that can account for place field formation and stabilization in order to understand the mechanisms underlying these processes. We therefore develop a data-driven model of the hippocampal CA1 network. We show that somatic disinhibition, together with spatially modulated inputs, is sufficient to form place fields. However, dendritic inhibition and synaptic plasticity allow for silent cells to turn into stable place cells. We show that the combined action of somatic and dendritic inhibition balances an increase in excitatory weights due to synaptic plasticity, so that place cells after exploration return to their baseline firing rate. Our model suggests that place cell stability is due to large excitatory synaptic weights and large dendritic inhibition. Therefore, our model suggests that different types of interneurons are essential to unravel the mechanisms underlying place field plasticity. Finally, we use our model to predict how to perturb place fields. Artificially induced dendritic events in place cells can shift place field location even after place field stabilization. Our model reproduces a wide range of observations from the hippocampal CA1 network, provides a circuit-level understanding, and finally makes predictions that can be tested in future experiments. Importantly, our model suggests that interneuron diversity is crucial for the emergence of place fields and their consolidation.

## Results

**Fig 1:**
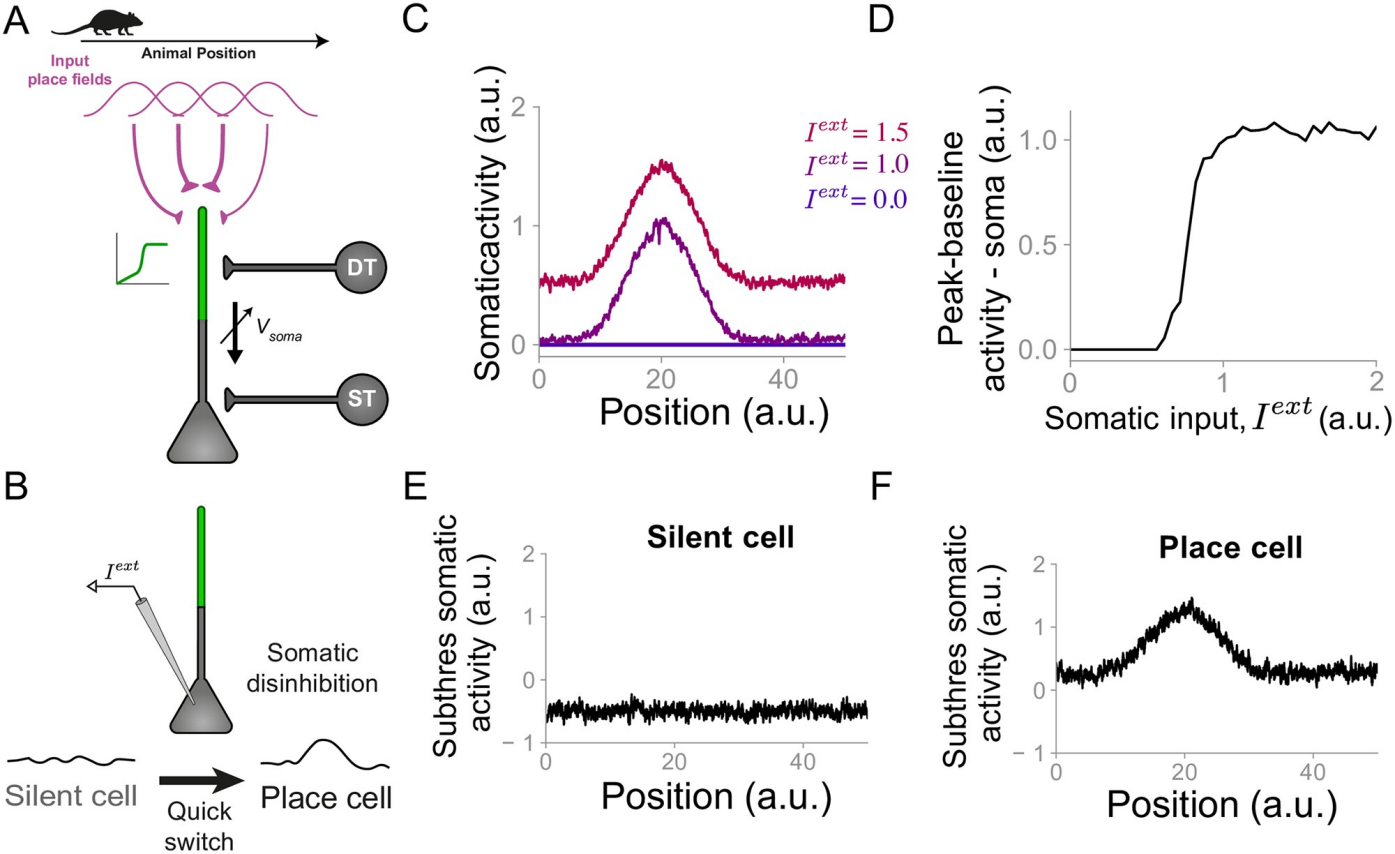
Somatic disinhibition is sufficient to transiently turn silents cells into place cells. **(A)** Network diagram. Pyramidal neurons receive place-tuned, excitatory input and inputs from two types of interneurons: dendrite-targeting (DT), representing somatostatin-expressing interneurons, and soma-targeting (ST), representing parvalbumin-expressing interneurons. The propagation of inputs from dendrites to soma is gated by the somatic “potential” (see Methods). The CA1 pyramidal cell is modelled as a two-compartment neuron model with a nonlinear dendritic unit and a perisomatic unit. **(B)** Diagram of a silent cell being turned into a place cell following spatially uniform somatic depolarization. The depolarization is induced by the injection of a constant current at the somatic compartment. **(C)** Pyramidal cell somatic activity as a function of the animal position for three different amplitudes of external injected current: zero, 1.0 and 1.5. **(D)** Difference between peak and baseline somatic activity as a function of the external somatic input. Because of the gated propagation of inputs from dendrites to soma, there is an abrupt transition from silent to place cell. **(E)** Subthreshold somatic activity (*V*_*soma*_) as a function of the animal position for a silent cell. Although the neuron is receiving place-tuned input onto its dendritic compartment, the neuronal subthreshold somatic activity is spatially uniform due the gated dendrite-to-soma propagation. **(F)** Subthreshold somatic activity (*V*_*soma*_) as a function of the animal position for a place cell.

**Fig 2:**
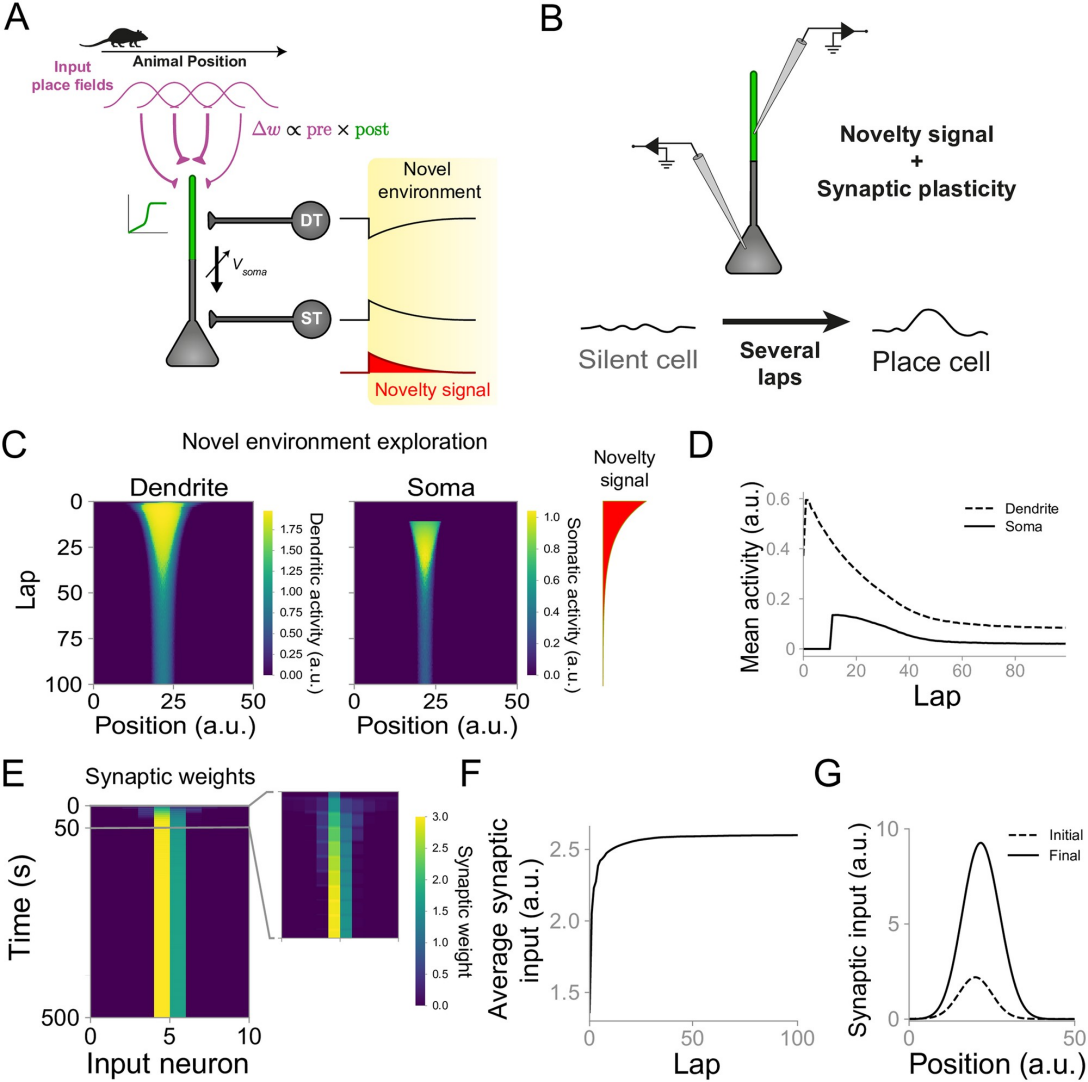
Dendritic disinhibition and synaptic plasticity promote the development of place cells. **(A)** Network diagram similar to Fig 1A. The activity of interneurons is modulated during the exploration of novel environments. DT interneuron activity (top black curve) decreases, whereas ST interneuron activity (bottom black curve) increases in novel environments. Both interneuron activities gradually return to baseline levels with a timescale defined by the hypothesized novelty signal (red curve, see Methods and main text for details). Synaptic connections from input neurons to CA1 pyramidal cells are updated following a Hebbian-type learning rule dependent on presynaptic activity and postsynaptic dendritic activation. **(B)** Diagram of a silent cell being turned into a place cell after several laps of exploration of a novel environment (see Methods). **(C)** Silent cell turns into place cell following exploration of a novel environment. Evolution of dendritic (left) and somatic (middle) activity during exploration of a novel environment for an initially silent cell. Amplitude of novelty signal over laps (right, red). Dendritic activity precedes somatic activation, in agreement with experiments [11]. Somatic activity increases abruptly due to the gated propagation of dendritic inputs (see Methods). **(D)** Evolution of mean dendritic and somatic activity for one example neuron. The neuron is initially silent (no somatic activity) and is turned into a place cell after several laps of exploration. **(E)** Evolution of synaptic weights for the same example cell shown in C. Inset: first 10% (50 s) of exploration. **(F)** Evolution of average synaptic input over laps for the same example cell as in C. **(G)** Initial (dashed) and final (solid) synaptic inputs as a function of the animal position for the same example cell as in C. The synaptic input was measured as the convolution between initial/final synaptic weights and the input neuron activities.

In all simulations, we model CA1 pyramidal neurons as two-compartment, rate-based neurons (Fig 1A), derived from a reduction of a detailed two-compartment spiking neuron model [57]. The neurons are composed of a non-linear dendritic unit, that accounts for dendritic spikes, and a perisomatic unit (Fig 1A). In our model, the activity (or rate) of these compartments can be related to either their spiking rate or a rectified version of its local voltage. In the hippocampal CA1 subregion, place cells can be observed even during the first stages of exploration of novel environments [10, 11, 13, 14, 19, 20, 21, 22, 23, 24, 25, 26, 27, 28] and silent cells can be quickly turned into place cells upon the injection of a spatially uniform current [12]. Therefore, we assume that all CA1 cells—both active and silent cells—receive place-tuned inputs which are projected onto their dendrites while the animal explores an environment. Additionally, the propagation of dendritic activity to the soma is not uniform and, in particular, can be modulated by somatic depolarization [12, 58]. In our model, the propagation of inputs from dendrites to soma is gated by the somatic “potential” which is determined by the total input projected directly onto the perisomatic unit (Fig 1A, see Methods). This gating does not block all the inputs to the perisomatic compartment, but only the inputs being propagated from the dendritic compartment. To account for changes in synaptic connections, we implement an activity-dependent synaptic plasticity rule. Synaptic potentiation has been shown to be dependent on the activation of presynaptic terminals paired with strong postsynaptic dendritic depolarization [7, 9, 15, 33, 37, 40]. For the sake of simplicity, we assume that synaptic plasticity depends on presynaptic activity and postsynaptic dendritic activation only (Fig 1A, see Methods for details). Moreover, the simulated CA1 pyramidal cells receive inhibitory inputs from two types of interneurons: dendrite-targeting interneurons (thought of as a subset of SST cells) and soma-targeting interneurons (thought of as a subset of parvalbumin-expressing cells) (Fig 1A). The interneuron activity is assumed to be spatially uniform [16]. Finally, Sheffield et al. [11] have shown that the exploration of novel environments modulates CA1 interneuron activity in an interneuron-type-specific manner [11]. They observed a decrease in SST interneuron activity accompanied by an increase in parvalbumin-expressing (PV) interneuron activity that lasts for tens of seconds when the animal enters a novel environment [11]. In our model, we hypothesize the existence of a novelty signal responsible for modulating interneuron activity once the animal enters a new environment (Fig 2A, see Methods). This novelty signal increases instantly once the animal enters the environment and decays exponentially with a time constant of 100 s (see Methods). This novelty signal leads to the suppression of dendrite-targeting inhibition and the amplification of soma-targeting inhibition (Fig 2A). Both interneuron activities slowly return to baseline levels as the novelty signal fades away.

### Somatic disinhibition is sufficient to transiently turn silent cells into place cells

We first investigate how silent cells can be transiently turned into place cells through the injection of a spatially uniform current [12]. We simulate 10 input neurons, which could be thought of as part of CA3, projecting onto one postsynaptic CA1 neuron. Here, we assume that the animal is running through a familiar environment and, therefore, there is no novelty signal and the interneuron activity is constant throughout these simulations. For simplicity, all presynaptic neurons are assumed to have time-invariant, uniformly distributed place fields, spanning over the entire track. These presynaptic neurons project onto one postsynaptic CA1 neuron through non-uniform connections. Although not uniform, the initial synaptic weights are such that the postsynaptic CA1 cell is silent during the first lap of exploration. During the second lap of exploration, an external, spatially uniform depolarizing current is applied to the somatic compartment. Since plasticity is slow, synaptic weights are not significantly changed from the first to the second lap. However, because the propagation of inputs from dendrites to soma is gated by somatic depolarization, silent cells are rapidly turned into place cells in an all-or-nothing manner (Fig 1B–1D). For weak external currents, silent cells remain silent (Fig 1C, *I*^*ext*^ = 0). For sufficiently strong external currents, however, silent cells are turned into place cells (Fig 1C, *I*^*ext*^ = 1.0 and *I*^*ext*^ = 1.5), in agreement with experiments [12]. Furthermore, the transition from silent to place cell is abrupt. Under the condition that the amplitude of the injected current is above a certain threshold, the neuron is turned into a place cell and the amplitude of the place field does not depend on the amplitude of the injected current (Fig 1D). If the external current is removed, the neuron becomes silent again. This all-or-nothing behavior is a direct consequence of the gated propagation of inputs from dendrites to soma. In our model, if this gating is removed, silent cells are instead gradually turned into place cells and the amplitude of its place field increases linearly with the external current (S1 Fig). The gating mechanism in our simulations does not prevent the perisomatic compartment from receiving inputs. Instead, the subthreshold somatic activity in silent cells fluctuates according to these inputs whereas place-tuned inputs arriving at the dendritic compartment are filtered (Fig 1E). Place cells, however, exhibit place-tuned subthreshold activity (Fig 1F). Therefore, our model indicates that silent cells can be transiently turned into place cells due to a combination of two features: silent cells receive place-tuned input and the propagation of these inputs from dendrites to soma is gated by somatic depolarization. Since the somatic input is also controlled by soma-targeting inhibitory neurons, these cells could also act as a gate for the propagation of inputs from dendrites to soma and therefore as a rapid mechanism to turn silent cells into place cells.

### Dendritic disinhibition and synaptic plasticity promote the development of place cells

Using our model, we next investigate whether there is an alternative mechanism underlying place field formation from originally silent cells (Fig 2A and 2B). As before, we simulate 10 input neurons projecting onto one postsynaptic CA1 neuron. Synaptic connections from input neurons to the postsynaptic neuron are plastic and their change depends on the activity of the postsynaptic dendritic compartment. Here, the animal explores a novel environment for several laps. Inspired by the experiments from Sheffield et al. [11], we simulate a “novelty signal”, so that the activity of dendrite-targeting interneurons is initially low in novel environments and increases gradually while the environment is becoming familiar, whereas the activity of soma-targeting interneurons is initially high and gradually decreases (Fig 2A). Simulating our model reveals that the reduction in dendrite-targeting inhibition increases dendritic activity in pyramidal cells, regardless of their somatic activity (Fig 2C). This dendritic activation leads to a quick strengthening in synaptic weights (Fig 2E–2G). The combination of stronger synaptic weights and a later decrease in soma-targeting inhibition finally leads to the development of place-tuned somatic activity (Fig 2C and 2D). Therefore, our model suggests that the combination of dendritic activity-dependent synaptic plasticity and novelty-modulated interneuron activity can turn silent cells into place cells. Interestingly, dendritic activity in simulated CA1 neurons precedes and predicts place field development in silent cells, consistent with the experimental findings from Sheffield et al. [11].

### The interplay between somatic and dendritic inhibition balances increased excitatory synaptic weights so that place cell firing returns to baseline

**Fig 3:**
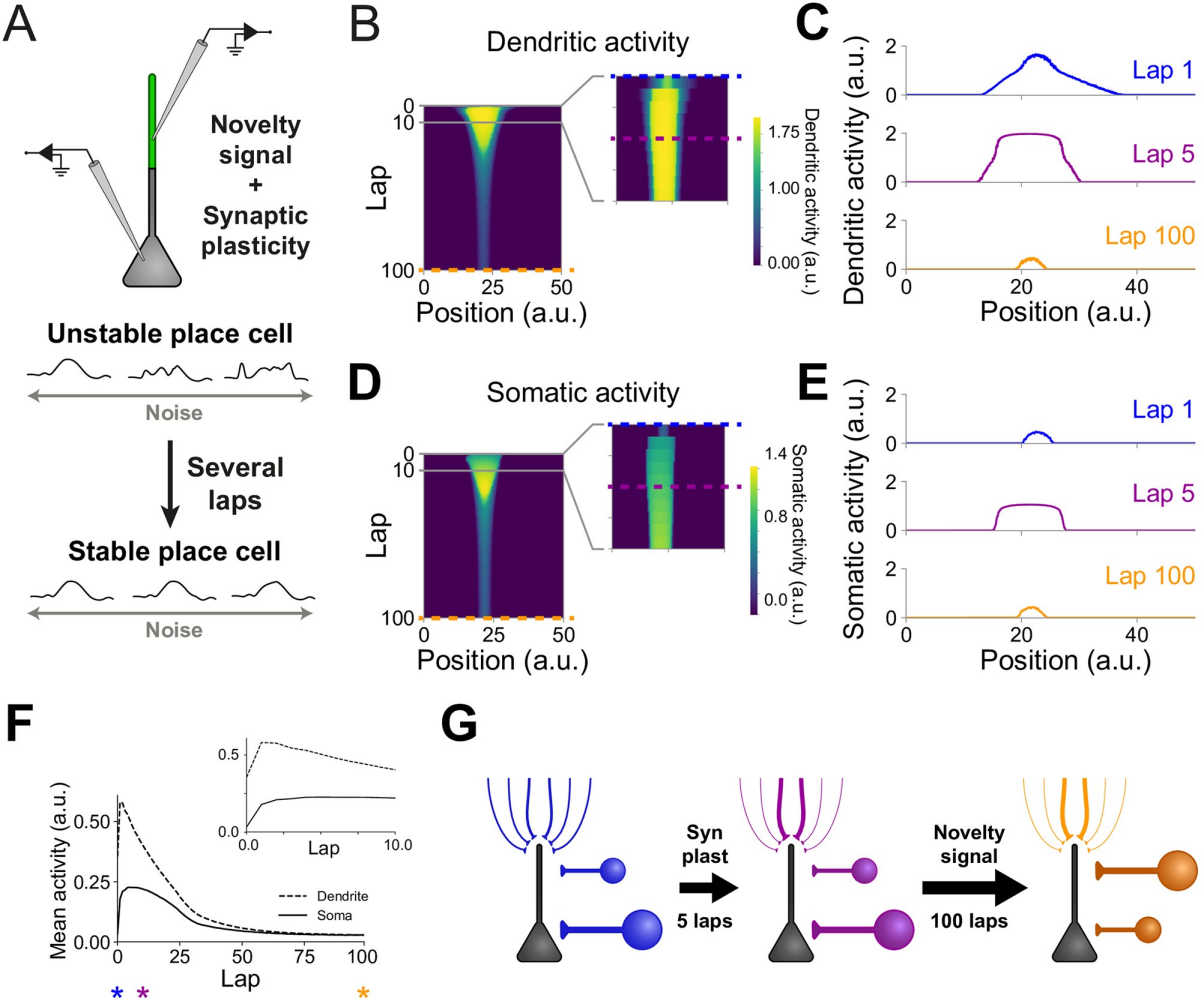
The interplay between somatic and dendritic inhibition balances increased excitatory synaptic weights so that place cell firing returns to baseline. **(A)** Diagram of an unstable place cell becoming stable after several laps of exploration of a novel environment (see Methods). Place cells are assumed to be unstable due to their sensitivity to noise. **(B)** Evolution of dendritic activity for an example place cell. Inset: first 10 laps of exploration. **(C)** Dendritic activity as a function of the animal’s position for three stages of the simulation: lap 1 (top, blue; blue dashed line in (B)), lap 5 (middle, purple; purple dashed line in (B)), and lap 100 (bottom, orange; orange dashed line in (B)). **(D)** Evolution of somatic activity for the same cell as in (B). Inset: first 10 laps of exploration. **(E)** Somatic activity as a function of the animal’s position for three stages of the simulation: lap 1 (top, blue; blue dashed line in (D)), lap 5 (middle, purple; purple dashed line in (D)), and lap 100 (bottom, orange; orange dashed line in (D)). **(F)** Evolution of mean dendritic (dashed line) and somatic (solid line) activity for the same example cell as in (B) and (D). Stars indicate laps 1 (blue), 5 (purple) and 100 (orange). Both somatic and dendritic activities increase sharply during the first laps of exploration due to synaptic plasticity. Inset: first 10 laps of exploration. **(G)** Diagram showing the changes in the network from the first to the last lap of exploration. Initially (left, blue), input synaptic weights are weak, dendritic inhibition is low and somatic inhibition is high. Next, synaptic weights are quickly strengthened through activity-dependent synaptic plasticity (middle, purple). During the final lap (right, orange), some input synaptic weights are strong, dendritic inhibition is high and somatic inhibition is low. Therefore, although place field amplitude and width are the same in the first and last lap (D blue and orange), the network is in a different state.

Next, we use our model to study neurons that are initially active when the animal enters a new environment (Fig 3A). We aim to understand the mechanisms that could lead to place field stabilization and that underlie place field dynamics. As before, our model consists of a CA1 cell receiving place-tuned inputs. But here, the synaptic weights are such that the neuron is active since the first lap of exploration (Fig 3B–3E, blue traces). The initially low dendritic inhibition in our model leads to the activation of dendritic compartments and thus the strengthening of synaptic weights. Stronger synaptic weights produce a stronger neuronal response (Fig 3B–3E, purple traces). However, as the animal explores the environment, the novelty signal gradually dissipates, resulting in the increase in dendritic inhibition and thus in a lower activation of the dendritic compartment (Fig 3C, orange trace). The lower level of somatic inhibition in familiar environments allows the neuron to exhibit the same level of activity as it exhibited during the first stages of exploration, even under reduced dendritic activation (Fig 3E, orange trace, and Fig 3F). Our model is therefore consistent with the experimental data showing that CA1 pyramidal cell depolarization increases in early stages of novel environment exploration and later returns to initial levels in familiar environments [10]. The novelty signal hypothesized in our model plays a key role in this behavior and removing it from our simulations leads to a higher neuronal activity in familiar rather than novel environments (S1 Fig). Importantly, although the neuronal firing on the first and last laps are indistinguishable, the network states are completely different. During the first lap, the neuron receives weak excitatory input, weak dendritic inhibition, and strong somatic inhibition (Fig 3G). Synaptic plasticity then leads to the strengthening of synaptic weights, forming strongly-tuned connections (Fig 3F and 3G). Finally, the decay of the novelty signal leads to a slow shift in soma- and dendrite-targeting inhibition. During the last lap—when the environment is familiar—the neuron receives strong excitatory input, strong dendritic inhibition, and weak somatic inhibition (Fig 3G). Therefore, the intricate interplay between excitatory plasticity and somatic and dendritic inhibition leads to the development of a new network structure. Although this new state presents the same network output, it might have different stability properties.

Although our simulations were performed using a simplified, rate-based neuron, the same results can be achieved using a biophysical, spatially extended neuron model (S2 Fig). In this case, a spiking neuron increases its firing rate over the first few laps of exploration of a novel environment which later returns to baseline level when the novelty signal fades away (S2 Fig). To further explore the robustness of our model, we rerun our simulations using our rate-based neuron but modifying specific aspects of the model. Firstly, we consider the case in which the novelty signal is also applied to the inputs, resulting in an initially higher place-tuned excitatory input. In this case, CA1 place fields evolve similarly to before (S3 Fig). Initial place fields, however, are stronger in these conditions (S3 Fig). Next, we modify the neuronal motif used in our simulations by including recurrent connections to account for feedback inhibition (S4 Fig). The feedback inhibition attenuates the amplitude of the final place field without affecting the qualitative behavior of place field evolution (S4 Fig). Finally, synaptic plasticity can be induced by non-coincident activity following a learning rule that spans over seconds [15]. In order to introduce this behavioral-time-scale plasticity in our model, we implemented a learning rule that potentiates synaptic weights for neurons that are active together within a time window in the order of seconds (S5 Fig, see S1 Methods). This learning rule results in a competition amongst a larger number of inputs, leading to initially wider place fields (S5 Fig). Nevertheless, the evolution of place fields follows a similar pattern as the one we observed with a simple hebbian learning rule. The amplitude of place fields is initially low and transiently increases before returning to a lower level (S5 Fig). Therefore, the results observed with our model are robust to more complex or extended models.

### Large excitatory synaptic weights and large dendritic inhibition provide place cell stability

**Fig 4:**
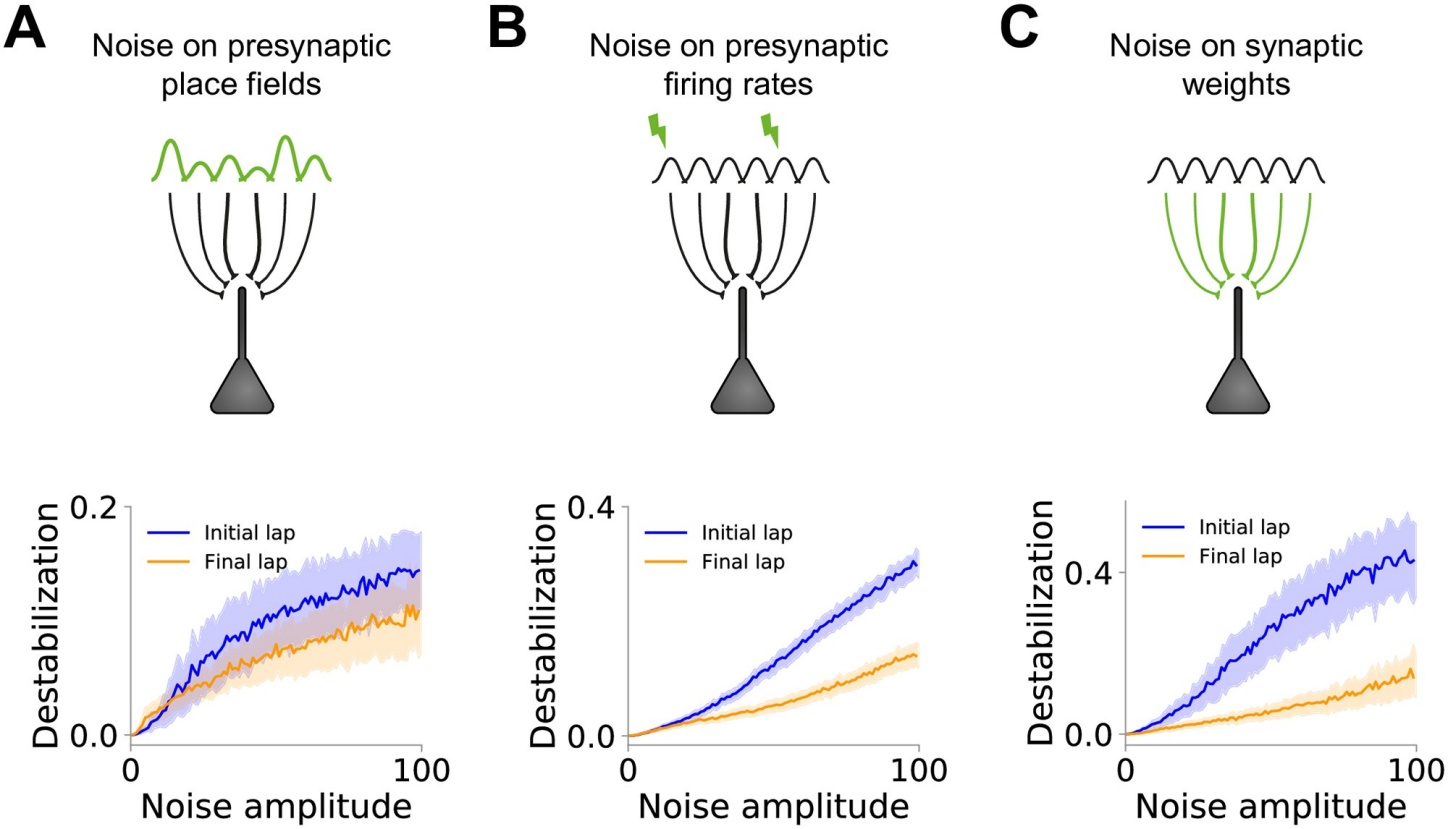
Large excitatory synaptic weights and large dendritic inhibition provide place cell stability. **(A-C)** Effect of noise on place fields for the first (blue) and last (orange) laps of exploration.**(A)** Destabilization of place fields by noise on presynaptic place fields. We measure the change on postsynaptic place field following changes on presynaptic place field amplitudes (see Methods). **(B)** Destabilization of place fields by noise on presynaptic firing rates. We measure the change on postsynaptic place field following the addition of a noisy input to presynaptic neurons (see Methods). **(C)** Destabilization of place fields by noise on synaptic weights. We measure the change in postsynaptic place field following changes on synaptic weights (see Methods). For all three sources of noise (A-C), the effect of the noise over place fields is higher in the first lap than in the last lap.

We next investigate whether place fields in familiar environments are more stable than at the beginning of the exploration phase in novel environments—despite being similar in amplitude and width. Place fields have been shown to be unreliable and to change abruptly from lap to lap in novel environments [10]. We speculate that this variability is caused by the place field’s sensitivity to noise rather than synaptic plasticity processes. We assume that the place field can be affected by three sources of noise: (i) noise on the place fields of presynaptic neurons, (ii) noise on the firing rates of presynaptic neurons, or (iii) noise on synaptic weights, accounting, for example, for synaptic turnover or synaptic failure (Fig 4). In all three cases, we compare the effect of noise on place fields at the beginning of exploration (Fig 4, blue curves) to its effect on place fields at the end of exploration (Fig 4, orange curves; see Methods). In case (i), we assume that the amplitudes of presynaptic place fields are not all the same. Instead, we multiply each place field by a random number whose variance increases with the noise amplitude (see Methods). As expected, the more noise we impose, the less stable place cells are (Fig 4A). However, the noise on presynaptic place fields is more effective at destabilizing place cells in the first lap of exploration than at the end of exploration (Fig 4A), suggesting that place cells become more stable. In case (ii), we assume that all presynaptic place fields have the same amplitude but input neurons can also fire at any time with probability *p*. This probability increases linearly with noise amplitude. Again, place fields at the final lap are more stable than initial place fields (Fig 4B). In case (iii), we change synaptic weights by random amounts drawn from a normal distribution whose variance is proportional to the noise amplitude. Once again, this source of noise has a stronger effect on place fields on initial laps of exploration (Fig 4C). In all three cases, the stabilization of place fields results from increased synaptic weights and higher dendritic inhibition (Fig 3G). Therefore, place fields in familiar environments are more stable to noise than place fields at the beginning of exploration of novel environments, consistent with experimental observations [10].

In order to investigate the role of each component of the network in stabilizing place fields, we artificially modify the final state of the network while keeping the neuron’s place field unchanged. We first reduce the amplitude of both excitatory weights and dendritic inhibition (S6A Fig). The reduced synaptic weights decrease place field stability when noise is added on the synaptic weights (S6B Fig). Next, we reduce dendritic inhibition and increase somatic inhibitory input (S6C Fig). Since synaptic weights are strong and dendritic inhibition is low, the postsynaptic neuron is more susceptible to presynaptic inputs. Thus, noise on presynaptic neurons is carried on to postsynaptic place fields, destabilizing them (S1D Fig). In summary, strong synaptic connections are relatively less affected by noise on synaptic weights, whereas higher dendritic inhibition cancels out-of-field fluctuations being transmitted from presynaptic neurons.

We next investigate whether dendritic nonlinearity can contribute to stable place field development. While our model has indicated that dendritic disinhibition opens a window for synaptic plasticity and promotes place cell development, we hypothesize that dendritic nonlinearities might promote place cell stability by ensuring that the location of a place field does not change once the place field is developed. In our model, when inputs are strong enough, they can induce dendritic spikes, which in turn lead to strong potentiation. Because of competition mechanisms such as synaptic normalization, the remaining inputs are depressed, pushing them further away from the threshold for dendritic events. Without dendritic nonlinearities, the noise could be enough to counter-balance this competition, leading to unstable place fields. As such, dendritic spikes—or dendritic nonlinearities—might form a mechanism for reliably selecting presynaptic inputs. To test this hypothesis, we simulate our model with initially uniform synaptic weights and no novelty signal. We then compare it with an alternative model where dendrites do not have an amplifying nonlinearity but can reach the same maximum level of activity (linear dendrites, S7A and S7B Fig). Neurons with a dendritic nonlinearity develop place fields faster and, importantly, more reliably (S7C Fig). In several cases, neurons with linear dendrites do not develop place fields and their activities vary from lap to lap (S7D Fig). Contrarily, neurons with a dendritic nonlinearity consistently develop stable place fields. Therefore, our model suggests that dendritic nonlinearities might contribute to place field development and stability by promoting a reliable selection of inputs.

### Artificially induced dendritic events induce place field plasticity

**Fig 5:**
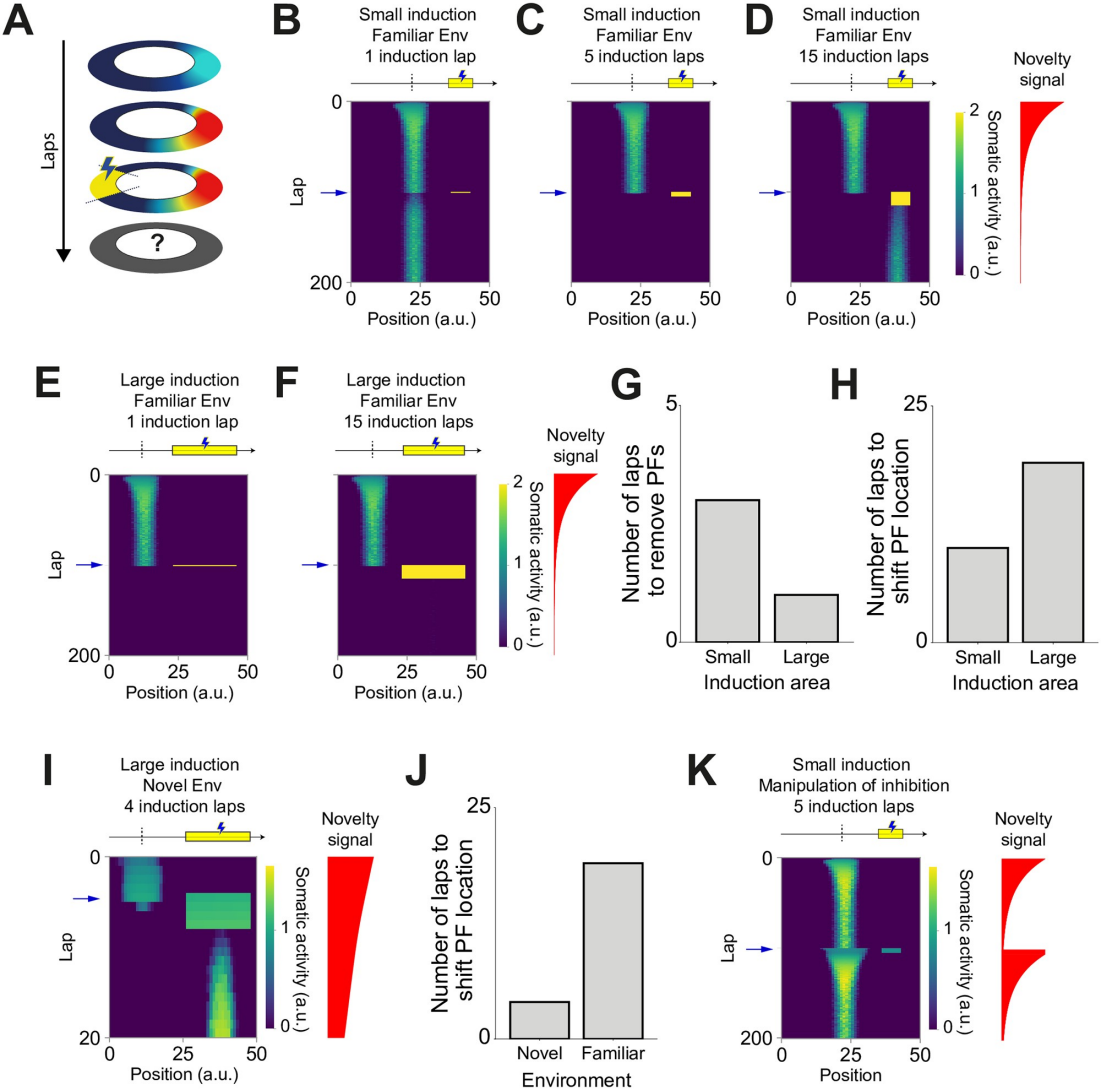
Artificially induced dendritic events induce place field plasticity. **(A)** Single-cell diagram. **(B-D)** Evolution of place fields for the case in which an extra current is applied to the postsynaptic neuron while the animal traverses a small section (15%) of the track. Yellow bar indicates the induction region in which the extra current is applied. Dashed line indicates the position of the peak of the initial place field. Blue arrow indicates the first induction lap (lap 100). Red curve shows the evolution of the novelty signal over laps. **(B)** Place field evolution for 1 induction lap. Place fields are not disturbed following the application of extra current. **(C)** Place field evolution for 5 induction laps. Place fields are removed by the application of extra current. **(D)** Place field evolution for 15 induction laps. Place fields are shifted towards a new position determined by the region of extra current application. **(E-F)** Evolution of place fields for the case in which an extra current is applied to the postsynaptic neuron while the animal traverses a large section (45%) of the track. Yellow bar indicates the induction region in which the extra current is applied. Dashed line indicates the position of the peak of the initial place field. Blue arrow indicates the first induction lap (lap 100). Red curve shows the evolution of the novelty signal over laps. **(E)** Place field evolution for 1 induction laps. Place fields are removed by the application of extra current. **(F)** Place field evolution for 15 induction laps. Place fields are removed by the application of extra current. **(G)** Number of induction laps required to remove stable place field for small and large induction areas. **(H)** Number of induction laps required to shift place field location for small and large induction areas. **(I)** Evolution of place fields for the case in which an extra current is applied during exploration of a novel environment (lap 5). The extra current is applied to the postsynaptic neuron while the animal traverses a large section (45%) of the track. Yellow bar indicates the induction region in which the extra current is applied. Dashed line indicates the position of the peak of the initial place field. Blue arrow indicates the first induction lap (lap 5). Red curve shows the evolution of the novelty signal over laps. **(J)** Number of induction laps required to shift place field location for novel and familiar environments. **(K)** Evolution of place fields for the case in which the application of an extra current is paired with the resetting of the novelty signal. The extra current is applied to the postsynaptic neuron while the animal traverses a small section (15%) of the track. Yellow bar indicates the induction region in which the extra current is applied. Dashed line indicates the position of the peak of the initial place field. Blue arrow indicates the first induction lap (lap 100). Red curve shows the evolution of the novelty signal over laps. Re-setting the novelty signal leads to a reduction in dendritic inhibition across the whole track. Therefore, the in-field activity increases, leading to the reinforcement of the initial place field.

Using our model, we next explore whether it is possible to perturb or change single CA1 place fields [15, 38, 40, 42]. We simulate a single neuron receiving place-tuned input such that one of its input synapses is stronger than the remaining connections. We assume that the animal is exploring a novel environment. As such, interneuron activity is modulated by a novelty signal that decays over time. The stronger synaptic weight leads to the activation of the postsynaptic neuron, which leads to the strengthening of that synaptic weight (Fig 5A and 5B, see Methods). This positive feedback loop leads to the development of a strong place field when the environment becomes familiar (Fig 5B).

We then test whether we can shift the tuning of the place field towards a new location by artificially activating CA1 neurons. In order to do that, we simulate the network until the novelty signal is negligible—the environment is hence considered familiar—and the postsynaptic place field is stable. At this stage, we inject an extra current in the dendritic compartment of the simulated neuron to induce a strong dendritic activity. The current is injected in the dendritic compartment because, in our model, the synaptic plasticity is assumed to depend on dendritic activation only. This current is induced only in a small region within the track, far from the peak of the postsynaptic place field (Fig 5, see Methods). The induction of extra dendritic activity over one lap does not alter the postsynaptic place field (Fig 5B). We next induce the extra dendritic activity over several (15) laps. In this case, the position of the place field is shifted towards the new location (Fig 5D). For an intermediate number of induction laps, the initial place field is removed without the formation of a new place field, thus turning the place cell into a silent cell (Fig 5C, S8C Fig). Note that this newly formed silent cell can potentially redevelop a place field in case there is remaining dendritic activity. This dendritic activity allows for plasticity, and therefore for the re-emergence of a place field (S3B Fig). Altogether, our model predicts that, if induced over enough laps, artificial dendritic activity can shift place field location.

The size of the induction region might affect the efficacy to shift place field location. We hypothesized that shifting place fields would be easier with a larger induction region. To investigate this, we increase the induction area to three times its original size. In this case, the induction over one lap is enough to remove the initial place field, but not enough to induce the formation of a new one (Fig 5E). In contrary to what we hypothesized, the induction over 15 laps—which is enough to induce the development of a new place field for a small induction area—is not enough to promote the development of a new place field (Fig 5F). The larger the induction area, the easier it is to remove the initial place tuning (Fig 5G). Nevertheless, a large induction area leads to a competition between inputs within that area. Because of that, our model predicts that, surprisingly, the larger the induction region, the more induction laps are needed to induce the development of new receptive fields (Fig 5H).

We next compare the induction of place field shift in novel and familiar environments. We hypothesize that in novel environments, place fields should be more plastic and, therefore, it should be easier to induce a shift in place field location. In order to test this, we induce dendritic activity on lap 5 instead of 100. As shown above, the induction protocol in familiar environments has to be applied over several laps to successfully induce place field shift. In novel environments, conversely, applying the induction protocol over a few laps is enough to induce the development of a new place field. Indeed, the induction of dendritic activity over 4 laps is sufficient to shift place field location (Fig 5I and 5J). We then compare the artificial induction of place field shift with the development of place fields following naturally occurring dendritic spikes (here detected as dendritic activity crossing a threshold for non-linear amplification, see S1 Methods and S1 Table). In our simulations, natural dendritic spikes need, on average, less than 2 laps to induce the development place fields whereas artificially induced dendritic spikes need at least 4 laps to induce a shift in receptive field location in novel environments (S8G Fig). Naturally occurring dendritic spikes are more efficient because they promote the potentiation of already strong synaptic weights, whereas artificially induced activity usually has to recruit weak connections. As initially hypothesized, our model indicates that we need fewer induction laps to induce place field shift in novel environments than in familiar ones. This extra plasticity of place fields in novel environments is due to two factors: synaptic weights are not yet strongly tuned in the first laps, and the novelty signal induces an increase in postsynaptic dendritic activity.

Finally, we investigate whether we can artificially manipulate the interneuron activity in familiar environments so that the model returns to the state it is found in novel environments. In particular, place fields could become more plastic following the manipulation of interneuron activity. To test that, we run the simulations for 100 laps—until the environment becomes familiar. At lap 100, we decrease dendrite-targeting inhibition and increase soma-targeting inhibition, resetting them to the level of novel environment exploration. Simultaneously, at lap 100, we induce dendritic activity within a region far from the peak of the neuron’s place field. Since the modulation of inhibition is applied over the entire environment, there is an increase in both within-field and out-of-field firing rate. Accordingly, the shift in place field location is harder than in the case without manipulation of inhibition (Fig 5K). Next, we suppress dendritic inhibition only when the animal is inside of a section of the environment, not overlapping with the neuron’s original place field, and without direct external stimulation onto the CA1 pyramidal cell (S8E and S8F Fig). This spatially-confined dendritic disinhibition leads to the shift place field location (S8E and S8F Fig). We conclude that, surprisingly, re-setting inhibition to novel environment levels is not enough to make place fields plastic again. Indeed, overall manipulation of inhibition reinforces stable place fields by increasing within-field activity. Spatially-restricted dendritic disinhibition, however, can shift place field location.

In summary, our model suggests that single-cell place fields can be shifted under the induction of dendritic activity. Our model predicts that small induction areas are more effective at inducing the development of new place fields. Induction in novel environments is also more efficient than in familiar ones. Counter-intuitively, resetting novel environment level of inhibition represses place field plasticity.

## Discussion

We propose a model of hippocampal CA1 place cells in which interneuron activity is modulated by novelty in an interneuron-type-dependent manner. Using our simulations, we identify the potential mechanisms underlying the evolution of place fields and the transition from silent to place cells in novel environments. During the initial stages of exploration of novel environments, dendrite-targeting inhibition is reduced whereas soma-targeting inhibition is increased. The reduction in dendritic inhibition opens a window for plasticity, leading to the formation and stabilization of receptive fields. We then show that place fields are more stable in familiar environments than in novel environments. Our simulations suggest that this extra stability is due to stronger synaptic weights and increased dendritic inhibition. Our model makes predictions on how to perturb place fields by dendritic activation. In our model, dendritic activation can shift place field location. We predict that this shift is easier if the dendritic activity is induced only within a small region of the environment, in the order of the size of presynaptic place field widths. We also predict that it is easier to induce place field shift in novel than in familiar environments. Our model, albeit simple, provides a mechanism for several features of the CA1 network and provides testable predictions.

The modulation of interneuron activity during exploration of novel environments is thought to be important for place field development and stabilization. Dendritic events, such as NMDA spikes and Ca^2+^ plateau potentials [11, 15, 29], have been implicated in the development of new place fields. Thus, the reduction in dendritic inhibition—due to a reduction in SST interneuron activity, for example—might be responsible for opening a window for plasticity by promoting these dendritic events. Reduced inhibition could unmask small input inhomogeneities, leading to the rapid emergence of place cells during the first stages of exploration of novel environments. These small inhomogeneities would then be amplified through synaptic plasticity. The role of increased somatic inhibition in novel environments, however, is less clear. Since soma-targeting interneurons receive inputs from local pyramidal cells, the increase in soma-targeting interneuron activity could be reflecting the increase in pyramidal cell activity. Somatic inhibition can also be responsible for regulating pyramidal cell activity to ensure that the overall level of excitatory activity is kept within a certain regime. In our simulations, we induced a quick switch between silent and place cells by injecting an excitatory current onto the perisomatic section of the CA1 pyramidal cell. Endogenously, this change in somatic input might be mediated by soma-targeting interneurons such as a subset of PV expressing cells. These cells would then act as a gate and could quickly reassign which cells become active and therefore choose which cells encode the relevant spatial information. Following this quick assignment of place cells, further mechanisms such as back-propagating action potential or increased dendritic inputs could consolidate the newly defined place map. This control by PV interneurons might be important to ensure the development of sparse and robust representations. Overall, this dendrite- and soma-specific regulation could be a mechanism to separate the learning process into two stages such that spatial representations are first developed within the hippocampus before being communicated back to cortex. Furthermore, the increase in soma-targeting interneuron activity can also be responsible for controlling plasticity at CA1 pyramidal neurons.

Our model provides several predictions that could be tested experimentally. In our simulations, place fields can be formed even in the absence of a novelty signal (S1A Fig). However, the dynamics of the evolution of place fields are altered in this scenario. For instance, the amplitude of newly developed place fields does not progress towards a lower level with spatial exploration (S1A Fig). Although controlling the novelty signal experimentally—by modulating both SST and PV interneuron activity simultaneously—would be extremely challenging, this prediction could be tested by analyzing place fields formed after several laps of exploration, when the novelty signal would be much weaker and the interneuron activity would have returned to its baseline level [11]. Furthermore, in our simulations, the quick and abrupt switch from silent to place cell is a direct consequence of the nonlinear propagation of inputs from dendrites to soma. This non-linearity has been shown to be mediated by a persistent sodium current [59]. In the absence of this gating mechanism, place field amplitude would vary across a wider range of amplitudes (S1B Fig). This could be tested by measuring place field amplitudes during the suppression of the persistent sodium current in single cells while the animal explored an environment [59]. Finally, our model provides specific prediction on the efficiency of perturbation protocols to shift place field location. Although place fields have been manipulated experimentally by single cell stimulation [42], our model provides a framework that can be used to designing further protocols.

The inclusion of the nonlinear dendrite-to-soma propagation in a more biologically detailed version of our model should not affect the qualitative results presented here since our model is agnostic to the specific mechanism underlying the non-linear dendrite-to-soma propagation. Our model only assumes this propagation to be dependent on the baseline somatic depolarization, in agreement with experiments [12, 59]. A possible extension of our model could involve the implementation of other forms of nonlinearities. A smoother non-linear dendrite-to-soma propagation would result in a smoother transition from silent to place cell, which can also be observed in our model with the inclusion of noise on excitatory inputs (Fig 1).

For some of our simulations, we considered variations of our model using more complex implementations such as a biophysical, spatially extended spiking neuron model, and a recurrent network to take into account feedback inhibition. A further extension of our model could include, for example, a combination of both. PV and SST interneurons have been shown to have specific roles within the hippocampal CA1 network [60]. While both PV and SST silencing increase pyramidal cell activity, SST silencing controls pyramidal cell bursting activity whereas PV silencing controls the timing of pyramidal cell spiking within the theta cycle [60]. Combining a biophysically detailed neuron model with recurrent connection would allow us to explore these aspects of the CA1 hippocampal network and the introduction of a novelty signal could unravel how these different roles emerge during exploration of novel environments.

Aside from direct dendritic disinhibition, place cells might be formed by alternative—probably complementary—mechanisms such as via neuromodulation. Several neuromodulators such as acetylcholine, noradrenaline, dopamine and serotonin, have been implicated in long-term synaptic plasticity [61, 62, 63, 64, 65, 66]. These neuromodulators are responsible for changing the functional state of the hippocampal CA1 network and they might be responsible for modulating interneuron activity when the animal faces novel experiences [67, 68, 69]. The action of neuromodulators, however, may have further implications such as an increase in pyramidal cell excitability or change in the plasticity rules governing glutamatergic synapses. The inclusion of these factors in future versions of our model could help to unravel further details of the mechanisms associated with place field development.

In our simulations, we considered the development of place fields as a consequence of plasticity at glutamatergic synapses mediated by disinhibition. Although inhibition evolved with time in our model, inhibitory plasticity was not taken into account. Previous computational models have shown that place fields can be developed through the interaction between excitatory and inhibitory plasticity [43]. In these models, only one type of interneuron was considered and place fields could be formed from homogeneously distributed place-tuned inputs [43]. Therefore, our model complements previous models by introducing interneuron diversity while assuming place-tuned inputs. More recently, synapses from SST and PV interneurons onto CA1 pyramidal cells have been shown to follow interneuron-type-specific learning rules [70]. A combination of these recent findings and the novelty signal described in our model could lead to further insights and predictions regarding place field development and stabilization. The novelty signal could be responsible for the global emergence of place fields whereas the interaction between excitatory and inhibitory plasticity could be responsible for their refinement.

Following synaptic plasticity, CA1 pyramidal cells might exhibit spatially-tuned activity in our model. The place fields displayed by these neurons are the result of a combination of place fields exhibited by upstream neurons. This combination is then processed by the CA1 neuron in three stages: dendritic nonlinearities, dendrite-to-soma nonlinear propagation, and somatic rectification. These nonlinearities form important processing steps and ultimately lead to the formation of silent and place cells. To simplify our results and analysis, we impose single-peaked, spatially-tuned activity on input neurons. CA1 pyramidal cells, however, are also subject to more complex inputs such as grid-like inputs from entorhinal cortex or from multi-peaked CA3 place cells. The incorporation of these complex inputs in our model would likely lead to more complex CA1 pyramidal cell place fields. In this case, both dendrites and soma could exhibit multi-peaked place-tuned activity, as observed experimentally [71]. Future extensions of our model could explore the effect of multi-peaked place-tuned inputs on place field plasticity and remapping.

Across all of our simulations, we assumed that CA1 pyramidal cells received place-tuned input while being agnostic to the source of this input. These neurons, however, receive inputs from both the hippocampus and cortex, which target different sections of the CA1 pyramidal neurons. Therefore, changes in the distribution of inhibitory inputs can alter the excitability of proximal and distal section of CA1 neurons [11] and ultimately select the main drive of CA1 pyramidal cell activity [70]. The incorporation of these different sources of inputs in our model could lead to more complex place field dynamics. The inclusion of different input could also improve the extrapolation of our model to other brain regions and provide predictions regarding the selection of feedforward and feedback inputs in primary sensory areas [72, 73] or principal cell activity following inhibitory reorganization in motor cortex [74].

In our model, we considered synaptic plasticity to be dependent on presynaptic activity and postsynaptic dendritic activation only. Additionally, we considered only direct dendrite to soma propagation. Although dendritic activation is necessary for NMDA-dependent synaptic potentiation, this activation can indeed be induced by local NMDA spikes [35, 36, 37, 75, 76] and plateau potentials [38, 39, 40], but also by back-propagating action potentials [7, 32, 33, 34]. Therefore, whereas somatic activation does not seem to be necessary for place field formation, it might also promote synaptic plasticity. It would be interesting to include soma-to-dendrite propagation in future extensions of our model and investigate the consequences of somatic activation to place field formation and stability.

CA1 pyramidal cell depolarization has been shown to increase rapidly following exposure to novel environments [10, 14]. As suggested by Cohen et al. [10], this increase is associated with increased excitatory inputs onto CA1 pyramidal cells in our model. Through exploration, pyramidal cell firing rate returns to baseline levels in familiar environments. This later reduction in place cell firing rate has been suggested to be associated with a reduction in excitatory input [10]. Conversely, inspired by data from Sheffield et al. [11], our model suggests that the return to baseline firing rate might be associated with the combined increase in dendritic inhibition and decrease in somatic inhibition, while excitatory inputs remain strong. This strong dendritic inhibition and strong dendritic excitation might lead to a local balanced state, giving rise to experimentally observed dynamics [77].

Our model indicates that the balance between strong excitatory input and the interplay between dendritic and somatic inhibition leads to place field stabilization in familiar environments. Synaptic plasticity, in our model, leads to the strengthening of within-field inputs and weakening of out-of-field inputs. The importance of plasticity for place field stabilization is corroborated by experiments in which NMDA receptors have been shown to be important for place field stabilization [22, 27, 28]. The increase in dendritic inhibition in familiar environments in our model induces a reduction in dendritic events and, thus, a reduction in plasticity-induced changes in place fields. Additionally, the combination of weak out-of-field inputs and strong dendritic inhibition leads to higher robustness to noise. Overall, place fields stabilize following the exploration of novel environments, in agreement with experiments [10]. Besides changes in excitatory and inhibitory inputs, dendritic nonlinearities might also contribute to place field development and stabilization. In the presence of noise, our model indicates that dendritic nonlinearities are crucial for reliable place field development. Therefore, our model offers a possible mechanism for place field stabilization and highlights the importance of interneuron diversity and the balance between strong excitatory and inhibitory inputs for this stabilization.

Our model provides a mechanistic understanding of the CA1 network. It reproduces a variety of observations, such as the dynamics of place fields during the exploration of novel and familiar environments. Furthermore, we demonstrate that place fields can be manipulated by artificial depolarization of CA1 pyramidal cells or by spatially-restricted dendritic disinhibition in our model.

## Methods

### Neuron model

We use two-compartment, rate-based neuron models (except for simulations shown in S2 Fig). This model was derived from a reduction of a detailed two-comparment spiking neuron model [57]. Each neuron is modeled as two compartments: one representing the perisomatic region and another representing the apical dendrites. The dendritic compartment’s activity, *r*_*dend*_, is determined by

$$\begin{array}{c} \tau_{0}\frac{dr_{dend}}{dt}=-r_{dend}+g_{dend}(p_{dend})\;\text{,} \end{array}$$

where *τ*_0_ is a time constant, and *p*_*dend*_ is the dendritic “potential” variable given by

$$\begin{array}{c} p_{dend}=\sum_{i}w_{i}R_{i}-I_{dend}+I_{dend}^{ext}\;\text{,} \end{array}$$ (2)

where *R*_*i*_ is the firing rate of neuron *i* in the presynaptic layer, *w*_*i*_ is the synaptic weight from a neuron in the presynaptic layer, *I*_*dend*_ is the input from dendrite-targeting interneurons—simulating SST interneuron inputs, and $I_{dend}^{ext}$ is an external current applied to the dendritic compartment. The function *g*_*dend*_ is a non-linear function of the input to the dendritic compartment given by

$$\begin{array}{c} g_{dend}\left(I\right)=\alpha_{1}{\left[\tanh(I/I_{0})\right]}_{+}+\alpha_{2}(\frac{1}{2}\left(\tanh\left(2\left(I-I_{0}\right)\right)+1\right))\;\text{,} \end{array}$$

where [⋅]_+_ denotes a rectification that sets negative values to zero, *α*_1_ controls the linear gain of the dendritic compartment, *α*_2_ controls the amplitude of the non-linear term associated with dendritic spikes, and *I*_0_ is proportional to the minimum input current necessary for the induction of a dendritic spike (S9A and S9B Fig).

Inspired by experimental evidence showing that the propagation of dendritic activity to the soma can depend on somatic depolarization [12, 58], we implement a gating mechanism in our model. Inputs from dendrites are propagated to the soma following a non-linear propagation function that depends on the somatic “potential” *V*_*soma*_ = *E*_*soma*_ − *I*_*soma*_, where $E_{soma}=E_{soma}^{int}+I_{soma}^{ext}$ is sum of the excitatory input onto the perisomatic compartment, $E_{soma}^{int}$, and the external current applied to the soma, $I_{soma}^{ext}$, and *I*_*soma*_ is the input from soma-targeting interneurons—simulating PV interneuron inputs. The activity of the somatic compartment, *r*_*soma*_, is given by

$$\begin{array}{c} \tau_{0}\frac{dr_{soma}}{dt}=-r_{soma}+{\left[g_{prop}(V_{soma})r_{dend}+E_{soma}-I_{soma}-N_{th}\right]}_{+}\;\text{,} \end{array}$$

where *N*_*th*_ is the threshold for somatic activation, and the non-linear dendrite-to-soma propagation function *g*_*prop*_ is given by

$$\begin{array}{c} g_{prop}\left(V_{soma}\right)=\{\begin{array}{cc} 0 & \text{if}\;V_{soma}\leq \theta_{prop}\\ 1 & \text{if}\;V_{soma}>\theta_{prop} \end{array}\;\text{,} \end{array}$$

where *θ*_*prop*_ is a threshold for dendrite-to-soma propagation. In our model, the activity (or rate) of each compartment can be related to either its rate or a rectified version of its local voltage. The external current onto the somatic compartment, $I_{soma}^{ext}$, is set to zero for all simulations except for simulations on Fig 1 and S1 Fig, in which cases this current is set to a constant value over one entire lap.

### Synaptic plasticity model

Synaptic plasticity in hippocampal CA1 neurons has been shown to be dependent on the activation of presynaptic terminals and strong postsynaptic dendritic depolarization [7, 9, 15, 33, 37, 40]. In our simulations, synaptic weights from input neurons onto CA1 neurons are plastic and depend on the activities of the presynaptic neuron, *r*_*j*_, and the dendritic compartment of the postsynaptic neuron, *r*_*dend*_, as a standard Hebbian term. We include a homeostatic term that takes into account the sum of all synaptic weights onto the postsynaptic neuron. The synaptic weight from input neuron *j* to the postsynaptic neuron *i*, *w*_*ij*_ is updated following

$$\begin{array}{c} \frac{dw_{ij}}{dt}=\eta_{ex}r_{dend}^{i}r_{j}-\eta_{homeo}(\sum_{j}w_{ij}-\theta_{homeo})\;\text{,} \end{array}$$

where *η*_*ex*_ is the learning rate of excitatory connections, *η*_*homeo*_ is the learning rate of the homeostatic term, and *θ*_*homeo*_ is a target homeostatic constant.

### Position-modulated inputs

The simulated CA1 neurons receive feedforward input from *N*_*pre*_ neurons. These input neurons are tuned to specific locations and their firing rates span over the entire environment. All the place fields of input neurons have the same tuning width, *σ*_*pre*_, and the same amplitude, *A*_*pre*_. We assume that the animal explores an annular track of length *L* with speed *v*. The firing rate of an input neuron with place field centered at *p*_0_ is

$$\begin{array}{c} r^{input}\left(p\right)=A_{pre}\exp(-\frac{d^{2}}{2\sigma_{pre}^{2}})\;\text{,} \end{array}$$ (1)

where *p* is the animal’s position, and *d* is the distance, along the track, between the animal’s position and the center of the place field.

### Novelty signal

The activity of PV and SST interneurons has been shown to be modulated by novelty [9]. When an animal enters a novel environment, SST interneuron activity decreases whereas PV activity increases [9]. Analogously, when simulating the exploration of a novel environment, we assume that interneuron activity changes over time in an interneuron-type specific manner. We define a quantity, named novelty signal, that modulates the interneuron activity

$$\begin{array}{c} n\left(t\right)=\exp\left(-t/\tau_{n}\right)\;\text{,} \end{array}$$

where *t* is the time measured from the start of exploration, and *τ*_*n*_ is a time constant. The dendritic and somatic inhibition are then given by

$$\begin{array}{c} I_{dend/soma}\left(t\right)=I_{dend/soma}^{\infty }-(I_{dend/soma}^{\infty }-I_{dend/soma}^{0})n\left(t\right)\;\text{,} \end{array}$$

where $I_{dend/soma}^{\infty }$ is the inhibitory input onto the dendrite/soma in familiar environments, and $I_{dend/soma}^{0}$ is the initial inhibitory input onto the dendrite/soma in novel environments. The initial level of dendritic inhibition is assumed to be lower than its level in familiar environments, $I_{dend}^{0}<I_{dend}^{\infty }$. The initial level of somatic inhibition is assumed to be higher than its level in familiar environments, $I_{soma}^{0}>I_{soma}^{\infty }$.

### Measuring place field stability

In Fig 4, we analyze the stability of place fields in the first and last lap of novel environment exploration. In order to measure the effect of noise in novel environments, we go through the following steps: (1) we take the network in the state it was at the beginning of lap 1; (2) we simulate one lap of exploration, without plasticity; (3) we measure the place field of the postsynaptic neuron; (4) we rescale this place field such that its peak is set to 1; (5) we change the state of the network by adding noise to it (see below); (6) we repeat (2)-(4); (7) we calculate the absolute distance between the two rescaled receptive fields; (8) we repeat (6)-(7) *N*_*noise*_ times and take an average over all samples (S9 Fig). To measure the effect of noise in familiar environments, we follow the same steps but using the state of the network at the beginning of the last lap (lap 100) in step (1).

We assume that place fields can be affected by three sources of noise: (i) noise at presynaptic place fields, (ii) noise at presynaptic firing rates, and (iii) noise at synaptic weights. In case (i), we multiply each presynaptic receptive field (Eq 1) by a random variable taken from a normal distribution with mean 1 and variance *N*^2^. In case (ii), we assume that each presynaptic neuron receives an extra input, independent of its receptive field, and not tuned to the animal’s position. This extra input is taken from a normal distribution with mean 0 and variance *N*^2^ and then rectified to admit only positive values. In case (iii), we add a random number to each synaptic weight. This random number is taken from a normal distribution with mean 0 and variance *N*^2^. In all three cases, we define *N* as the noise amplitude.

### Parameters and simulations

All simulations were implemented in python and will be made available at ModelDB. The parameters used in our simulations can be found in Table 1.

**Table 1:** Parameters summary.

**Neuron Model**		
**Name**	**Value**	**Description**
*τ*_0_	5.0 ms	Firing rate time constant
*α*_1_	4/3	Linear gain of dendritic compartment
*α*_2_	2/3	Related to the amplitude of dendritic spikes
*I*_0_	2.5	Minimum current to induce dendritic spikes
*N*_*th*_	1.0	Threshold for somatic activation
*θ*_*prop*_	- 0.2	Threshold for dendrite-to-soma propagation
**Plasticity Model**		
**Name**	**Value**	**Description**
*η*_*ex*_	2 × 10^−4^ ms^−1^	Excitatory plasticity learning rate
*η*_*homeo*_	2 × 10^−4^ ms^−1^	Homeostatic plasticity learning rate
*θ*_*homeo*_	3.0 (Fig 5: 2.0)	Homeostatic target value
**Place-tuned input**		
**Name**	**Value**	**Description**
*A*_*pre*_	2.2	Presynaptic place field amplitude
*σ*_*pre*_	5.0	Presynaptic place field width
**Novelty signal**		
**Name**	**Value**	**Description**
*τ*_*n*_	100 s	Time constant for novelty signal decay
$I_{dend}^{0}$	0.8	Initial dendritic inhibition
$I_{dend}^{\infty }$	7.5 (fig2); 8.5 (fig3 & 5)	Target dendritic inhibition
$I_{soma}^{0}$	1.2	Initial somatic inhibition
$I_{soma}^{\infty }$	0.0	Target somatic inhibition
**Simulation parameters**		
**Name**	**Value**	**Description**
*N*_*pre*_	10	Number of presynaptic neurons
$E_{soma}^{int}$	0.0 (fig1); 0.5 (fig2); 1.0 (fig3 & 5)	Excitatory current onto perisomatic compartment
*T*_*length*_	50 a.u.	Track length (arbitrary units)
*v*	1 × 10^−2^ ms^−1^	Animal speed
*dt*	1 ms	Integration time step

## Supporting information

S1 Fig (related to Figs 1 and 3). Novelty signal and dendrite-to-soma propagation gating are required to reproduce experimental data. **(A)** Mean somatic (solid like) and dendritic (dashed line) activity as a function of the lap of exploration for a simulated CA1 pyramidal cell in which both dendritic and somatic inhibition were kept constant (no novelty signal) throughout the simulation. Mean activity increases within a few laps of exploration but does not return to baseline levels in familiar environments. **(B)** Left: pyramidal cell somatic activity as a function of the animal position for three different amplitudes of external injected current: zero, 1.0 and 1.5. Right: Difference between peak and baseline somatic activity as a function of the external somatic input. In these simulations, inputs from dendrites to the soma could propagate freely, without any gating mechanism. The gating mechanism is therefore essential for the abrupt transition from silent to place cell observed experimentally.
(PDF)

S2 Fig (related to Fig 3). The interplay between somatic and dendritic inhibition balances excitatory synaptic plasticity in a biophysical neuron model. **(A)** Simulated biophysical, spatially extended neuron modelled as a ball-and-stick neuron. The neuron receives place-tuned excitatory inputs and dendritic inhibition at the tip of a cylindrical compartment and somatic inhibition directly to the spherical, somatic compartment. Excitatory inputs are plastic and follow a Hebbian-type plasticity rule that depends on the amplitude of the excitatory input and the timing of postsynaptic spikes. Dendritic and somatic inhibition evolve in time following the novelty signal used in our rate-based simulations, i.e. dendritic inhibition increases over time whereas somatic inhibition decays. See supplementary S1 Methods for more details. **(B)** Mean firing rate over laps of exploration for simulated CA1 neurons. The average was calculated across 50 CA1 neurons and the shaded area represents the s.e.m. over all cells. Analogously to the results observed with rate-based neurons, the firing rate increases quickly over the first few laps and slowly returns to baseline level. **(C)** Firing rate as a function of the animal position for lap 1 (left), lap 5 (middle), and lap 80 (right). Firing rates were calculated as an average over 50 simulated CA1 cells under the same initial conditions. Place fields for laps 1 and 80 are similar whereas the place field at lap 5 is higher in amplitude. **(D)** Membrane voltage as a function of time across the first lap of exploration measured at the tip of the dendrite (top) and at the soma (bottom). **(E)** Membrane voltage as a function of time across the 80^th^ lap of exploration measured at the tip of the dendrite (top) and at the soma (bottom).
(PDF)

S3 Fig (related to Fig 3). Novelty signal at input neurons widens initial place fields without disturbing their dynamics. **(A)** Network diagram. Similar to simulations shown in Fig 2 with the introduction of novelty signal at input neurons. Pyramidal neurons receive place-tuned, excitatory input and inputs from two types of interneurons: dendrite-targeting (DT), representing somatostatin-expressing interneurons, and soma-targeting (ST), representing parvalbumin-expressing interneurons. The propagation of inputs from dendrites to soma is gated by the somatic “potential” (see Methods). The CA1 pyramidal cell is modelled as a two-compartment neuron model with a nonlinear dendritic unit and a perisomatic unit. The activity of interneurons is modulated during the exploration of novel environments. DT interneuron activity (top black curve) decreases, whereas ST interneuron activity (bottom black curve) increases in novel environments. Both interneuron activities gradually return to baseline levels with a timescale defined by the hypothesized novelty signal (red curve, see Methods and main text for details). The input neurons receive an extra input representing the effect of a novelty signal onto the input neurons. This extra current decays in time following the same time course as the novelty signal applied to inhibitory neurons. Synaptic connections from input neurons to CA1 pyramidal cells are updated following a Hebbian-type learning rule dependent on presynaptic activity and postsynaptic dendritic activation. **(B)** Evolution of mean dendritic (dashed line) and somatic (solid line) activity for one example cell. Both somatic and dendritic mean activities increase slightly during the first lap of exploration due to synaptic plasticity. **(C)** Evolution of dendritic activity for the same cell as in (B). Inset: first 10 laps of exploration. **(D)** Dendritic activity as a function of the animal’s position for three stages of the simulation: lap 1 (top, blue; blue dashed line in (C)), lap 5 (middle, purple; purple dashed line in (C)), and lap 100 (bottom, orange; orange dashed line in (C)). **(E)** Evolution of somatic activity for the same cell as in (B). Inset: first 10 laps of exploration. The peak somatic activity increases in the first few laps of exploration due to synaptic plasticity even though the mean somatic activity does not necessarily increase. **(F)** Somatic activity as a function of the animal’s position for three stages of the simulation: lap 1 (top, blue; blue dashed line in (E)), lap 5 (middle, purple; purple dashed line in (E)), and lap 100 (bottom, orange; orange dashed line in (E)).
(PDF)

S4 Fig (related to Fig 3). Feedback inhibition suppresses final place field amplitude while conserving place field evolution dynamics. **(A)** Network diagram. An extra connection from pyramidal cells to dendrite-targeting interneurons is introduced. the remaining network, including connectivity, novelty signal, and plasticity rules are identical to the ones implemented in Fig 2. The parameter *w*^*IE*^ is the synaptic weight for the connection from the pyramidal neuron to the dendrite-targeting interneuron (see supplementary S1 Methods). **(B)** Evolution of dendritic activity for *w*^*IE*^ = 0.5 (left), *w*^*IE*^ = 2.0 (middle), and *w*^*IE*^ = 5.0 (right). **(C)** Evolution of somatic activity for *w*^*IE*^ = 0.5 (left), *w*^*IE*^ = 2.0 (middle), and *w*^*IE*^ = 5.0 (right).
(PDF)

S5 Fig (related to Fig 3). Behavioral-time-scale plasticity promotes competition amongst a higher number of inputs while preserving place field dynamics. **(A)** Diagram learning window for behavioral-time-scale plasticity (BTSP). We implement a symmetric learning window with time constant *τ*. The change in synaptic weights depends on the activity of input neurons and the activity of the postsynaptic dendritic compartment (see supplementary S1 Methods). **(B)** Evolution of synaptic weights for one example cell with *τ* = 1.5 s (left) and *τ* = 1.0 s (right). Initial synaptic weights are chosen to slightly favor input neuron 4. Due to the long time window for plasticity, a higher number of input neurons compete to develop a postsynaptic place field. **(C)** Top: evolution of dendritic activity over 100 laps of exploration (left) and for the first 10 laps of exploration (right) for the same example cell in B left (*τ* = 1.5 s). Bottom: evolution of somatic activity over 100 laps of exploration (left) and for the first 10 laps of exploration (right). **(D)** Top: evolution of dendritic activity over 100 laps of exploration (left) and for the first 10 laps of exploration (right) for the same example cell in B right (*τ* = 1.0 s). Bottom: evolution of somatic activity over 100 laps of exploration (left) and for the first 10 laps of exploration (right).
(PDF)

S6 Fig (related to Fig 4). Strong synaptic weights and stronger dendritic inhibition ensures place field stability. **(A-B)** Strong synaptic weights provide stability to noise on synaptic connections. **(A)** Left: Network diagram for the network state at the last lap of exploration in Fig 4. Right: Modified network with reduced synaptic weights and reduced dendritic inhibition. Importantly, the changes are determined such that the neuron’s place field is kept unchanged. **(B)** Destabilization of place fields by noise on synaptic weights for final lap of exploration (orange) and modified network as in (B) (black). **(C)** Left: Network diagram for the network state at the last lap of exploration in Fig 4. Right: Modified network with reduced dendritic inhibition and increased somatic inhibition. Importantly, the changes are determined such that the neuron’s place field is kept unchanged. **(D)** Destabilization of place fields by noise on presynaptic firing rates for final lap of exploration (orange) and modified network as in (C) (black).
(PDF)

S7 Fig Dendritic non-linearity leads to reliable place field development. **(A)** Single-cell diagram. A pyramidal neuron receives input *I* and integrates it through a function *g*_*dend*_. **(B)** Dendritic transformation function *g*_*dend*_ as a function of the input *I* for linear dendrite (left, red) and nonlinear dendrites (right, green). **(C)** Spatial correlation between laps for blocks of 10 laps on simulations with nonlinear dendrites (green) and linear dendrites (red). Thick lines show averages over 200 cells for each group. Thin lines are individual cells. Note that the spatial correlation for several cells with linear dendrites does not increase over lap blocks. **(D)** Examples of individual pyramidal cells with linear dendrites. Top, evolution of neuron firing rate over laps as a function of the animal position. Middle, average neuron firing rate over the last 10 laps of exploration as a function of the animal position. Spatial correlation between laps for blocks of 10 laps. **(E)** Examples of individual pyramidal cells with nonlinear dendrites. Top, evolution of neuron firing rate over laps as a function of the animal position. Middle, average neuron firing rate over the last 10 laps of exploration as a function of the animal position. Spatial correlation between laps for blocks of 10 laps.
(PDF)

S8 Fig (related to Fig 5). Artificially induced CA1 single cell activity can shift place field location. **(A-D)** Evolution of place fields for the case in which an extra current is applied to the postsynaptic neuron while the animal traverses a section of the track. Yellow bar indicates the induction region in which the extra current is applied. Dashed line indicates the position of the peak of the initial place field. Blue arrow indicates the first induction lap. Red curve shows the evolution of the novelty signal over laps. **(A)** Place field evolution for 10 induction laps and small induction region (15% of the track). Place fields are shifted towards new position determined by the region of extra current application. **(B)** Place field evolution for 2 induction laps and small induction region (15% of the track). Place fields are transiently removed by the application of extra current and reemerge at the initial location. **(C)** Place field evolution for 3 induction lap and small induction region (15% of the track). Place fields are removed following the application of extra current. **(D)** Same as Fig 5I for a larger number of laps. Place field evolution for 5 induction laps and large induction region (45% of the track). The induction protocol is applied on lap 5, while the novelty signal is still strong. Place fields are shifted to new location. **(E-F)** Evolution of place fields for a simulation in which dendritic inhibition is suppressed while the animal traverses a section of the track. The disinhibition is induced only after the initial place field has been developed and the amplitude of the novelty signal is negligible. Blue bar indicates the induction region in which dendritic inhibition is suppressed. Dashed line indicates the position of the peak of the initial place field. Blue arrow indicates the first induction lap. Red curve shows the evolution of the novelty signal over laps. **(F)** Place field evolution for 5 induction laps. Place fields are removed following the suppression protocol. **(G)** Place field evolution for 15 induction laps. Place fields are shifted towards new position.
(PDF)

S9 Fig Dendritic non-linearity and stability analysis procedure. **(A)** Single-cell diagram. A pyramidal neuron receives input *I* and integrates it through a function *g*_*dend*_. **(B)** Diagram of *g*_*dend*_ as a function of the input *I* (see Methods). *α*_1_ controls the linear gain of the dendritic compartment; *α*_2_ controls the amplitude of the non-linear term related to dendritic spikes; and *I*_0_ controls the minimum input to elicit dendritic spikes. **(C)** Place field stability analysis. For each measurement of place field stability (see Methods) we perform the following steps: (i) we simulate one lap of exploration, without plasticity; (ii) we measure the place field of the postsynaptic neuron; (iii) we rescale this place field such that its peak is set to 1; (iv) we change the state of the network by adding noise to it; (v-vi) we repeat (ii)-(iii); (vii) we calculate the absolute distance between the two rescaled receptive fields.
(PDF)

S1 Methods Description of all the methods implemented in the simulations used to generate the supplementary figures. (PDF)

S1 Table Table of parameter values used in the simulations described in the supplementary methods. (PDF)
