# Supplementary material for: The interplay between somatic and dendritic inhibition promotes the emergence and stabilization of place fields
Source: PLoS Comput Biol. 2020 Jul 10;16(7):e1007955. doi: 10.1371/journal.pcbi.1007955 (PMC7386595; doi:10.1371/journal.pcbi.1007955)
Supplement: S1 Fig — (A) Mean somatic (solid like) and dendritic (dashed line) activity as a function of the lap of exploration for a simulated CA1 pyramidal cell in which both dendritic and somatic inhibition were kept constant (no novelty signal) throughout the simulation. Mean activity increases within a few laps of exploration but does not return to baseline levels in familiar environments. (B) Left: pyramidal cell somatic activity as a function of the animal position for three different amplitudes of external injected current: zero, 1.0 and 1.5. Right: Difference between peak and baseline somatic activity as a function of the external somatic input. In these simulations, inputs from dendrites to the soma could propagate freely, without any gating mechanism. The gating mechanism is therefore essential for the abrupt transition from silent to place cell observed experimentally. (PDF) [file pcbi.1007955.s001.pdf]

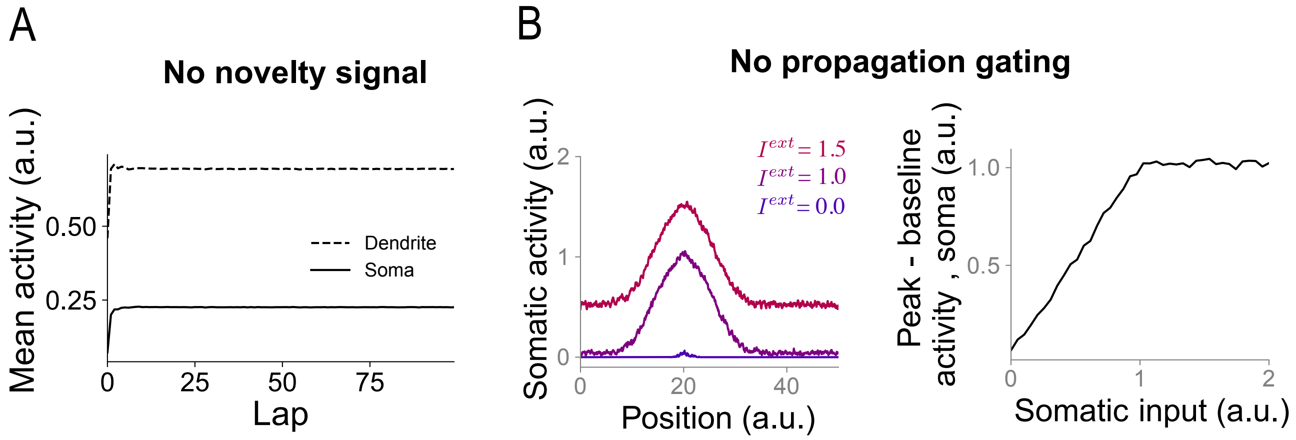

Figure S1 (related to figures 1 and 3). Novelty signal and dendrite-to-soma propagation gating are required to reproduce experimental data. (A) Mean somatic (solid like) and dendritic (dashed line) activity as a function of the lap of exploration for a simulated CA1 pyramidal cell in which both dendritic and somatic inhibition were kept constant (no novelty signal) throughout the simulation. Mean activity increases within a few laps of exploration but does not return to baseline levels in familiar environments. **(B)** Left: pyramidal cell somatic activity as a function of the animal position for three different amplitudes of external injected current: zero, 1.0 and 1.5. Right: Difference between peak and baseline somatic activity as a function of the external somatic input. In these simulations, inputs from dendrites to the soma could propagate freely, without any gating mechanism. The gating mechanism is therefore essential for the abrupt transition from silent to place cell observed experimentally.
