# Supplementary material for: The interplay between somatic and dendritic inhibition promotes the emergence and stabilization of place fields
Source: PLoS Comput Biol. 2020 Jul 10;16(7):e1007955. doi: 10.1371/journal.pcbi.1007955 (PMC7386595; doi:10.1371/journal.pcbi.1007955)
Supplement: S2 Fig — (A) Simulated biophysical, spatially extended neuron modelled as a ball-and-stick neuron. The neuron receives place-tuned excitatory inputs and dendritic inhibition at the tip of a cylindrical compartment and somatic inhibition directly to the spherical, somatic compartment. Excitatory inputs are plastic and follow a Hebbian-type plasticity rule that depends on the amplitude of the excitatory input and the timing of postsynaptic spikes. Dendritic and somatic inhibition evolve in time following the novelty signal used in our rate-based simulations, i.e. dendritic inhibition increases over time whereas somatic inhibition decays. See supplementary S1 Methods for more details. (B) Mean firing rate over laps of exploration for simulated CA1 neurons. The average was calculated across 50 CA1 neurons and the shaded area represents the s.e.m. over all cells. Analogously to the results observed with rate-based neurons, the firing rate increases quickly over the first few laps and slowly returns to baseline level. (C) Firing rate as a function of the animal position for lap 1 (left), lap 5 (middle), and lap 80 (right). Firing rates were calculated as an average over 50 simulated CA1 cells under the same initial conditions. Place fields for laps 1 and 80 are similar whereas the place field at lap 5 is higher in amplitude. (D) Membrane voltage as a function of time across the first lap of exploration measured at the tip of the dendrite (top) and at the soma (bottom). (E) Membrane voltage as a function of time across the 80th lap of exploration measured at the tip of the dendrite (top) and at the soma (bottom). (PDF) [file pcbi.1007955.s002.pdf]

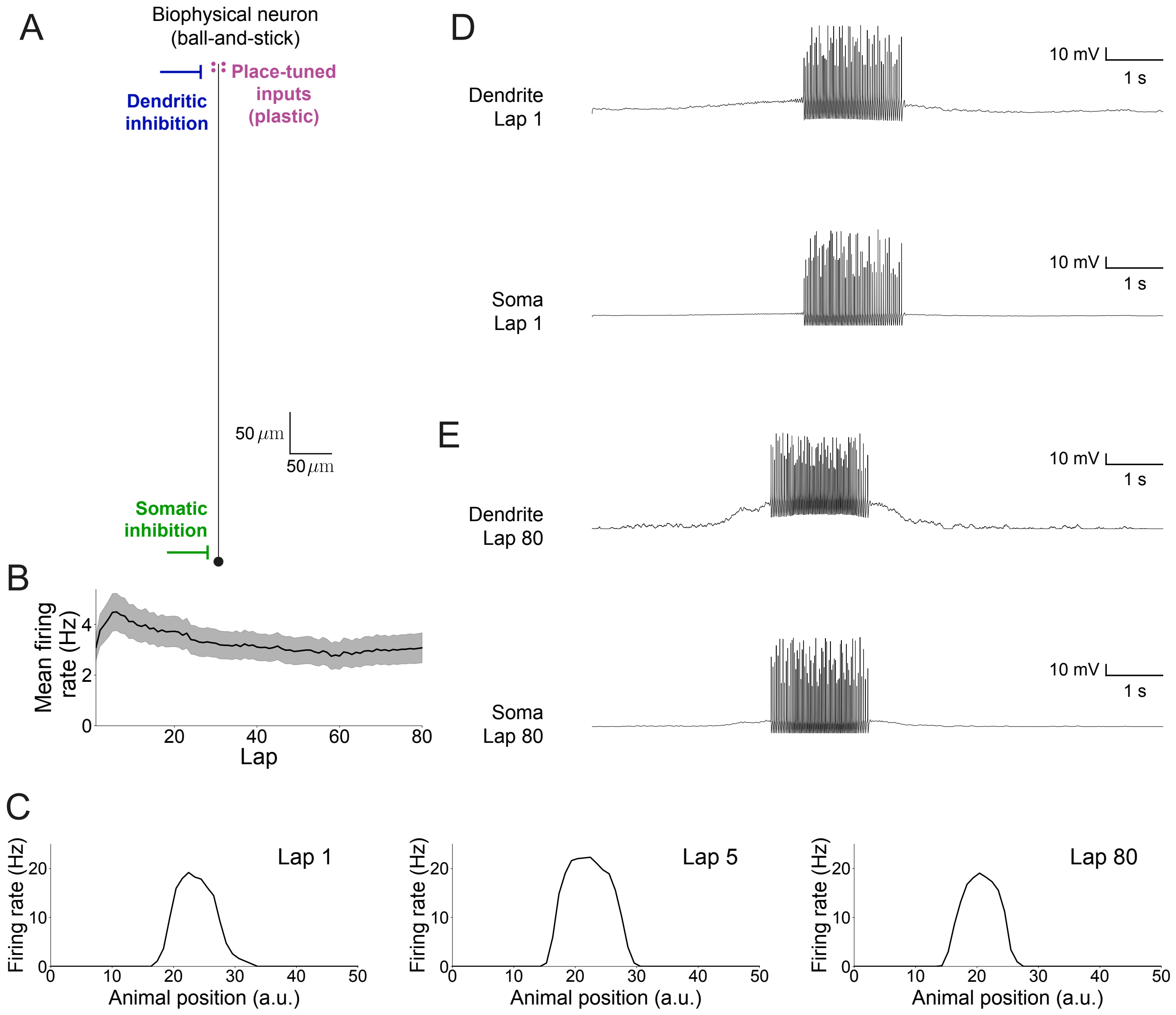

Figure S2 (related to figure 3). The interplay between somatic and dendritic inhibition balances excitatory synaptic plasticity in a biophysical neuron model. **(A)** Simulated biophysical, spatially extended neuron modelled as a ball-and-stick neuron. The neuron receives place-tuned excitatory inputs and dendritic inhibition at the tip of a cylindrical compartment and somatic inhibition directly to the spherical, somatic compartment. Excitatory inputs are plastic and follow a Hebbian-type plasticity rule that depends on the amplitude of the excitatory input and the timing of postsynaptic spikes. Dendritic and somatic inhibition evolve in time following the novelty signal used in our rate-based simulations, i.e. dendritic inhibition increases over time whereas somatic inhibition decays. See supplementary methods for more details. **(B)** Mean firing rate over laps of exploration for simulated CA1 neurons. The average was calculated across 50 CA1 neurons and the shaded area represents the s.e.m. over all cells. Analogously to the results observed with rate-based neurons, the firing rate increases quickly over the first few laps and slowly returns to baseline level. **(C)** Firing rate as a function of the animal position for lap 1 (left), lap 5 (middle), and lap 80 (right). Firing rates were calculated as an average over 50 simulated CA1 cells under the same initial conditions. Place fields for laps 1 and 80 are similar whereas the place field at lap 5 is higher in amplitude. **(D)** Membrane voltage as a function of time across the first lap of exploration measured at the tip of the dendrite (top) and at the soma (bottom). **(E)** Membrane voltage as a function of time across the 80th lap of exploration measured at the tip of the dendrite (top) and at the soma (bottom).
