# Supplementary material for: The interplay between somatic and dendritic inhibition promotes the emergence and stabilization of place fields
Source: PLoS Comput Biol. 2020 Jul 10;16(7):e1007955. doi: 10.1371/journal.pcbi.1007955 (PMC7386595; doi:10.1371/journal.pcbi.1007955)
Supplement: S3 Fig — (A) Network diagram. Similar to simulations shown in Fig 2 with the introduction of novelty signal at input neurons. Pyramidal neurons receive place-tuned, excitatory input and inputs from two types of interneurons: dendrite-targeting (DT), representing somatostatin-expressing interneurons, and soma-targeting (ST), representing parvalbumin-expressing interneurons. The propagation of inputs from dendrites to soma is gated by the somatic “potential” (see Methods). The CA1 pyramidal cell is modelled as a two-compartment neuron model with a nonlinear dendritic unit and a perisomatic unit. The activity of interneurons is modulated during the exploration of novel environments. DT interneuron activity (top black curve) decreases, whereas ST interneuron activity (bottom black curve) increases in novel environments. Both interneuron activities gradually return to baseline levels with a timescale defined by the hypothesized novelty signal (red curve, see Methods and main text for details). The input neurons receive an extra input representing the effect of a novelty signal onto the input neurons. This extra current decays in time following the same time course as the novelty signal applied to inhibitory neurons. Synaptic connections from input neurons to CA1 pyramidal cells are updated following a Hebbian-type learning rule dependent on presynaptic activity and postsynaptic dendritic activation. (B) Evolution of mean dendritic (dashed line) and somatic (solid line) activity for one example cell. Both somatic and dendritic mean activities increase slightly during the first lap of exploration due to synaptic plasticity. (C) Evolution of dendritic activity for the same cell as in (B). Inset: first 10 laps of exploration. (D) Dendritic activity as a function of the animal’s position for three stages of the simulation: lap 1 (top, blue; blue dashed line in (C)), lap 5 (middle, purple; purple dashed line in (C)), and lap 100 (bottom, orange; orange dashed line in (C)). (E) Evolutio [file pcbi.1007955.s003.pdf]

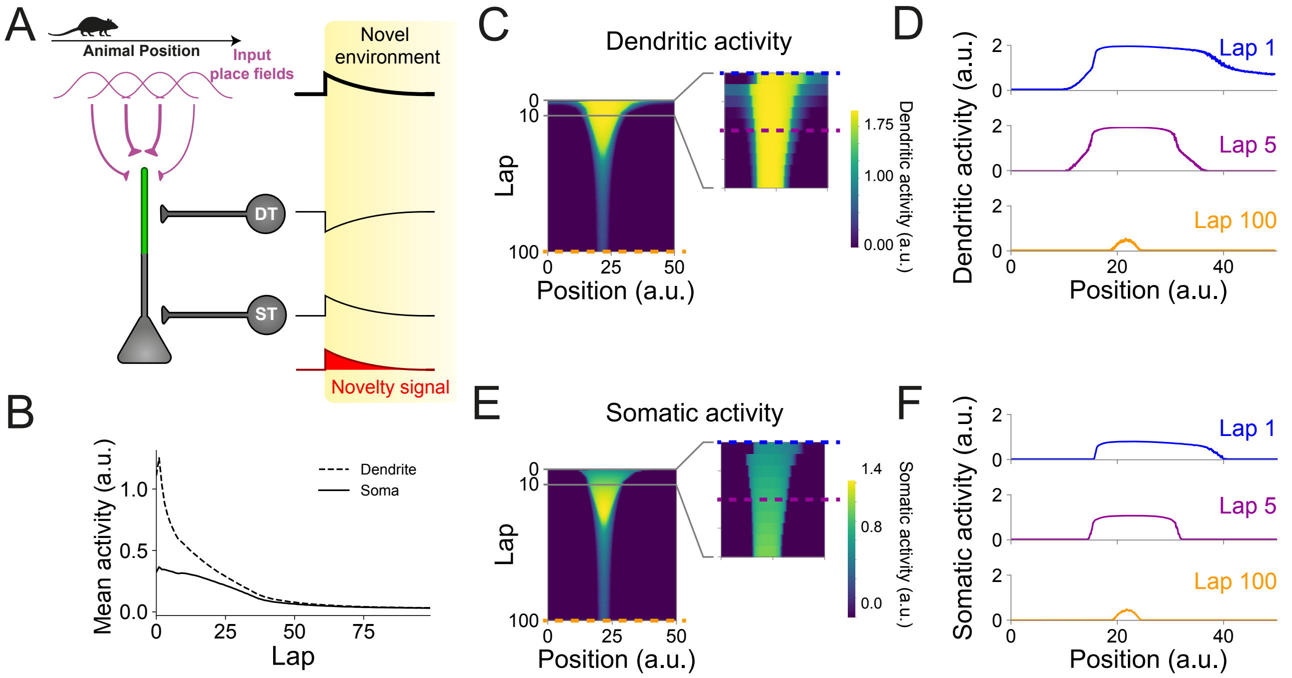

Figure S3 (related to figure 3). Novelty signal at input neurons widens initial place fields without disturbing their dynamics. (A) Network diagram. Similar to simulations shown in figure 2 with the introduction of novelty signal at input neurons. Pyramidal neurons receive place-tuned, excitatory input and inputs from two types of interneurons: dendrite-targeting (DT), representing somatostatin-expressing interneurons, and soma-targeting (ST), representing parvalbumin-expressing interneurons. The propagation of inputs from dendrites to soma is gated by the somatic "potential" (see methods). The CA1 pyramidal cell is modelled as a two-compartment neuron model with a nonlinear dendritic unit and a perisomatic unit. The activity of interneurons is modulated during the exploration of novel environments. DT interneuron activity (top black curve) decreases, whereas ST interneuron activity (bottom black curve) increases in novel environments. Both interneuron activities gradually return to baseline levels with a timescale defined by the hypothesized novelty signal (red curve, see methods and main text for details). The input neurons receive an extra input representing the effect of a novelty signal onto the input neurons. This extra current decays in time following the same time course as the novelty signal applied to inhibitory neurons. Synaptic connections from input neurons to CA1 pyramidal cells are updated following a Hebbian-type learning rule dependent on presynaptic activity and postsynaptic dendritic activation. **(B)** Evolution of mean dendritic (dashed line) and somatic (solid line) activity for one example cell. Both somatic and dendritic mean activities increase slightly during the first lap of exploration due to synaptic plasticity. **(C)** Evolution of dendritic activity for the same cell as in (B). Inset: first 10 laps of exploration. **(D)** Dendritic activity as a function of the animal's position for three stages of the simulation: lap 1 (top, blue; blue dashed line in (C)), lap 5 (middle, purple; purple dashed line in (C)), and lap 100 (bottom, orange; orange dashed line in (C)). **(E)** Evolution of somatic activity for the same cell as in (B). Inset: first 10 laps of exploration. The peak somatic activity increases in the first few laps of exploration due to synaptic plasticity even though the mean somatic activity does not necessarily increase. **(F)** Somatic activity as a function of the animal's position for three stages of the simulation: lap 1 (top, blue; blue dashed line in (E)), lap 5 (middle, purple; purple dashed line in (E)), and lap 100 (bottom, orange; orange dashed line in (E)).
