# Supplementary material for: The interplay between somatic and dendritic inhibition promotes the emergence and stabilization of place fields
Source: PLoS Comput Biol. 2020 Jul 10;16(7):e1007955. doi: 10.1371/journal.pcbi.1007955 (PMC7386595; doi:10.1371/journal.pcbi.1007955)
Supplement: S4 Fig — (A) Network diagram. An extra connection from pyramidal cells to dendrite-targeting interneurons is introduced. the remaining network, including connectivity, novelty signal, and plasticity rules are identical to the ones implemented in Fig 2. The parameter wIE is the synaptic weight for the connection from the pyramidal neuron to the dendrite-targeting interneuron (see supplementary S1 Methods). (B) Evolution of dendritic activity for wIE = 0.5 (left), wIE = 2.0 (middle), and wIE = 5.0 (right). (C) Evolution of somatic activity for wIE = 0.5 (left), wIE = 2.0 (middle), and wIE = 5.0 (right). (PDF) [file pcbi.1007955.s004.pdf]

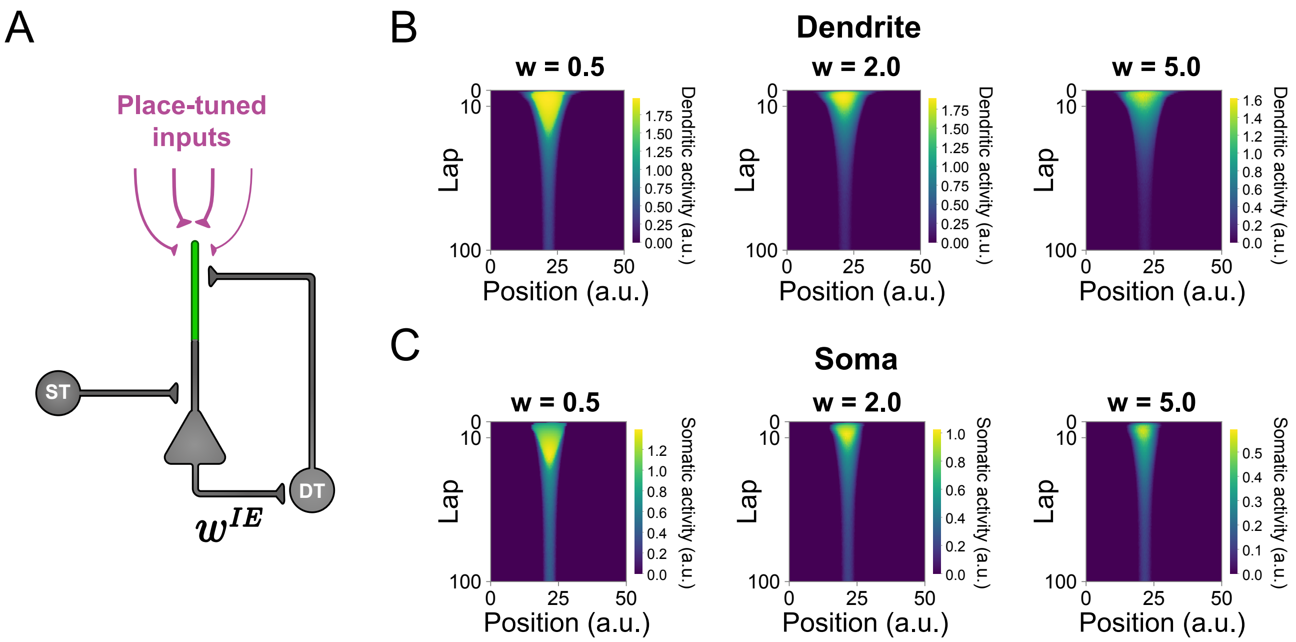

Figure S4 (related to figure 3). Feedback inhibition suppresses final place field amplitude while conserving place field evolution dynamics. **(A)** Network diagram. An extra connection from pyramidal cells to dendrite-targeting interneurons is introduced. the remaining network, including connectivity, novelty signal, and plasticity rules are identical to the ones implemented in figure 2. The parameter w^{IE} is the synaptic weight for the connection from the pyramidal neuron to the dendrite-targeting interneuron (see supplementary methods). **(B)** Evolution of dendritic activity for $w^{IE} = 0.5$ (left), $w^{IE} = 2.0$ (middle), and $w^{IE} = 5.0$ (right). **(C)** Evolution of somatic activity for $w^{IE} = 0.5$ (left), $w^{IE} = 2.0$ (middle), and $w^{IE} = 5.0$ (right).
