# Supplementary material for: The interplay between somatic and dendritic inhibition promotes the emergence and stabilization of place fields
Source: PLoS Comput Biol. 2020 Jul 10;16(7):e1007955. doi: 10.1371/journal.pcbi.1007955 (PMC7386595; doi:10.1371/journal.pcbi.1007955)
Supplement: S5 Fig — (A) Diagram learning window for behavioral-time-scale plasticity (BTSP). We implement a symmetric learning window with time constant τ. The change in synaptic weights depends on the activity of input neurons and the activity of the postsynaptic dendritic compartment (see supplementary S1 Methods). (B) Evolution of synaptic weights for one example cell with τ = 1.5 s (left) and τ = 1.0 s (right). Initial synaptic weights are chosen to slightly favor input neuron 4. Due to the long time window for plasticity, a higher number of input neurons compete to develop a postsynaptic place field. (C) Top: evolution of dendritic activity over 100 laps of exploration (left) and for the first 10 laps of exploration (right) for the same example cell in B left (τ = 1.5 s). Bottom: evolution of somatic activity over 100 laps of exploration (left) and for the first 10 laps of exploration (right). (D) Top: evolution of dendritic activity over 100 laps of exploration (left) and for the first 10 laps of exploration (right) for the same example cell in B right (τ = 1.0 s). Bottom: evolution of somatic activity over 100 laps of exploration (left) and for the first 10 laps of exploration (right). (PDF) [file pcbi.1007955.s005.pdf]

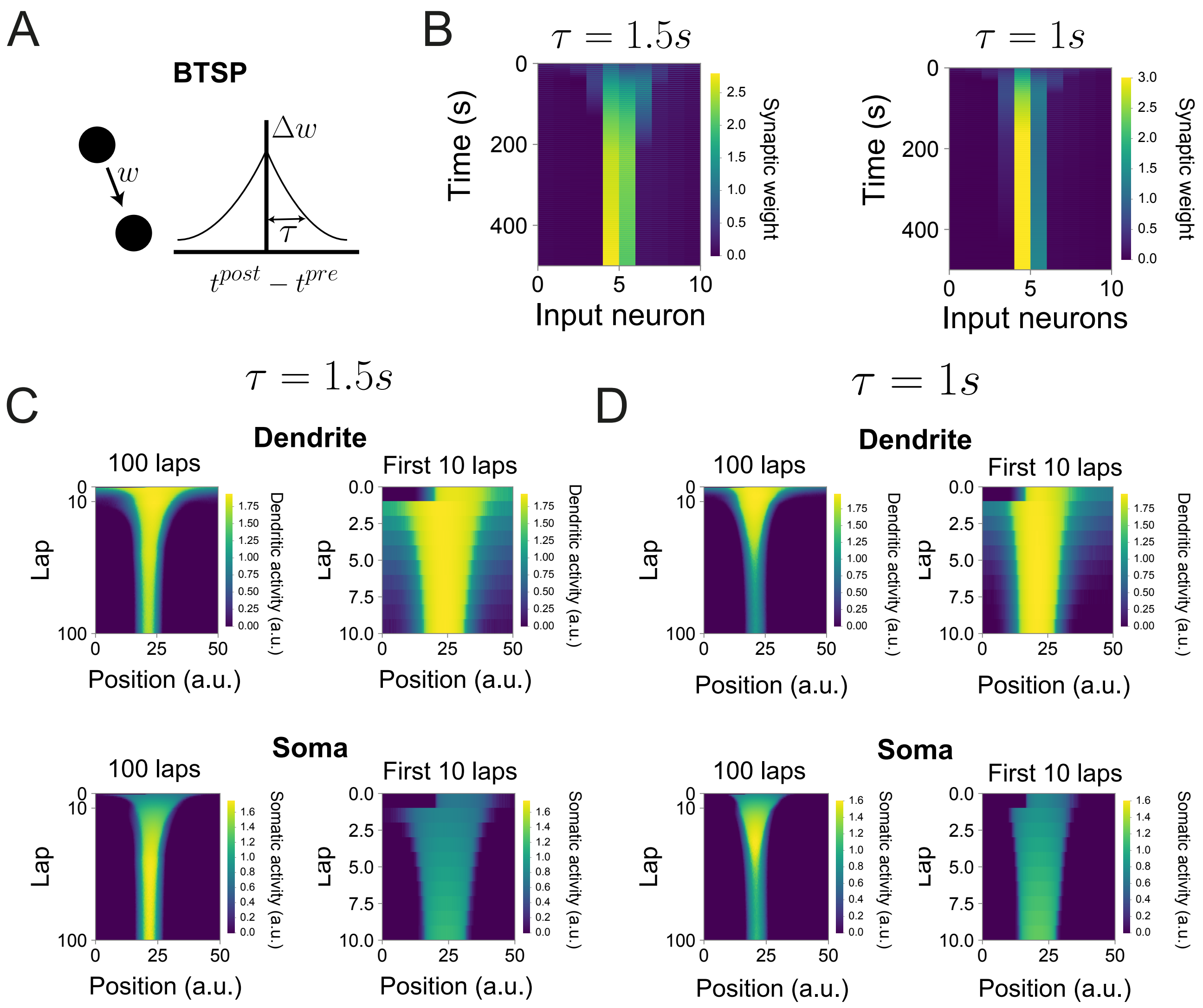

Figure S5 (related to figure 3). Behavioral-time-scale plasticity promotes competition amongst a higher number of inputs while preserving place field dynamics. **(A)** Diagram learning window for behavioral-time-scale plasticity (BTSP). We implement a symmetric learning window with time constant τ. The change in synaptic weights depends on the activity of input neurons and the activity of the postsynaptic dendritic compartment (see supplementary methods). **(B)** Evolution of synaptic weights for one example cell with $\tau = 1.5$ s (left) and $\tau = 1.0$ s (right). Initial synaptic weights are chosen to slightly favor input neuron 4. Due to the long time window for plasticity, a higher number of input neurons compete to develop a postsynaptic place field. **(C)** Top: evolution of dendritic activity over 100 laps of exploration (left) and for the first 10 laps of exploration (right) for the same example cell in B left ($\tau = 1.5$ s). Bottom: evolution of somatic activity over 100 laps of exploration (left) and for the first 10 laps of exploration (right). **(D)** Top: evolution of dendritic activity over 100 laps of exploration (left) and for the first 10 laps of exploration (right) for the same example cell in B right ($\tau = 1.0$ s). Bottom: evolution of somatic activity over 100 laps of exploration (left) and for the first 10 laps of exploration (right).
