# Supplementary material for: The interplay between somatic and dendritic inhibition promotes the emergence and stabilization of place fields
Source: PLoS Comput Biol. 2020 Jul 10;16(7):e1007955. doi: 10.1371/journal.pcbi.1007955 (PMC7386595; doi:10.1371/journal.pcbi.1007955)
Supplement: S6 Fig — (A-B) Strong synaptic weights provide stability to noise on synaptic connections. (A) Left: Network diagram for the network state at the last lap of exploration in Fig 4. Right: Modified network with reduced synaptic weights and reduced dendritic inhibition. Importantly, the changes are determined such that the neuron’s place field is kept unchanged. (B) Destabilization of place fields by noise on synaptic weights for final lap of exploration (orange) and modified network as in (B) (black). (C) Left: Network diagram for the network state at the last lap of exploration in Fig 4. Right: Modified network with reduced dendritic inhibition and increased somatic inhibition. Importantly, the changes are determined such that the neuron’s place field is kept unchanged. (D) Destabilization of place fields by noise on presynaptic firing rates for final lap of exploration (orange) and modified network as in (C) (black). (PDF) [file pcbi.1007955.s006.pdf]

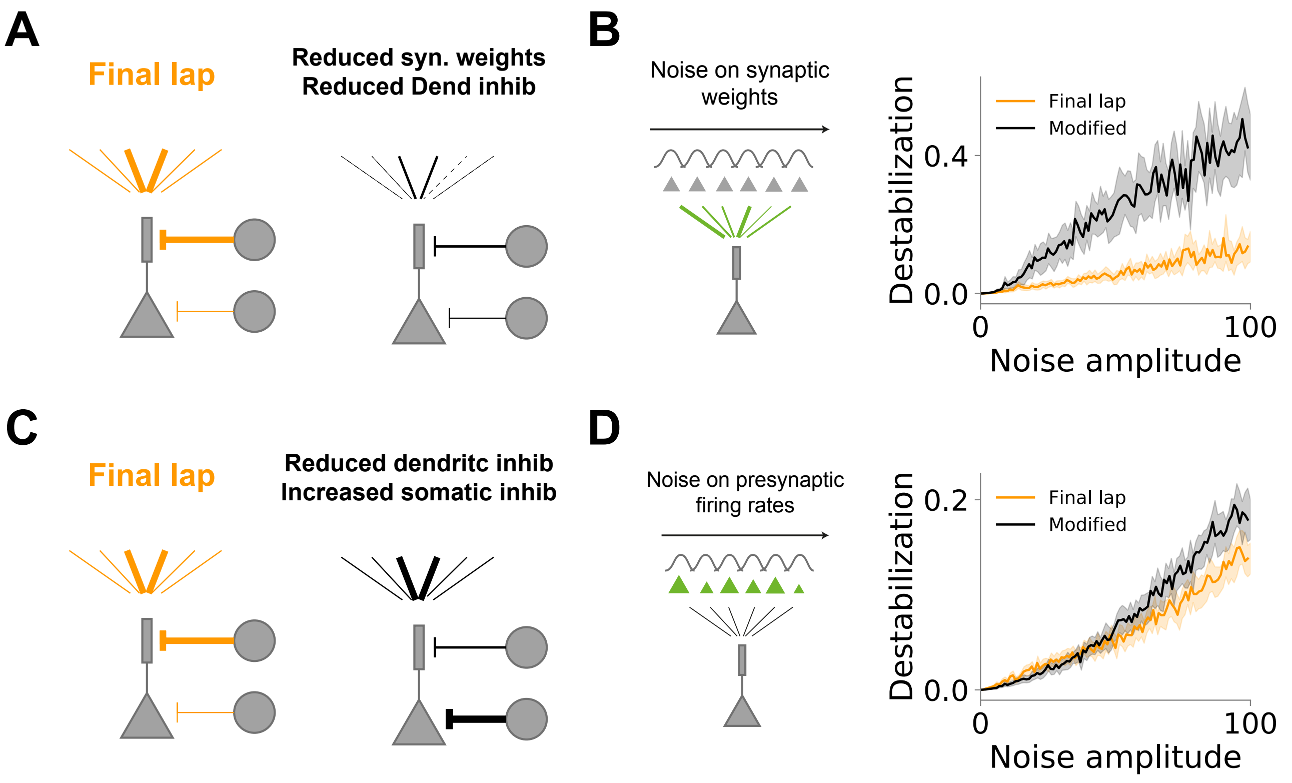

Figure S6 (related to figure 4). Strong synaptic weights and stronger dendritic inhibition ensures place field stability. (A-B) Strong synaptic weights provide stability to noise on synaptic connections. **(A)** Left: Network diagram for the network state at the last lap of exploration in figure 4. Right: Modified network with reduced synaptic weights and reduced dendritic inhibition. Importantly, the changes are determined such that the neuron's place field is kept unchanged. **(B)** Destabilization of place fields by noise on synaptic weights for final lap of exploration (orange) and modified network as in (B) (black). **(C)** Left: Network diagram for the network state at the last lap of exploration in figure 4. Right: Modified network with reduced dendritic inhibition and increased somatic inhibition. Importantly, the changes are determined such that the neuron's place field is kept unchanged. **(D)** Destabilization of place fields by noise on presynaptic firing rates for final lap of exploration (orange) and modified network as in (C) (black).
