# Supplementary material for: The interplay between somatic and dendritic inhibition promotes the emergence and stabilization of place fields
Source: PLoS Comput Biol. 2020 Jul 10;16(7):e1007955. doi: 10.1371/journal.pcbi.1007955 (PMC7386595; doi:10.1371/journal.pcbi.1007955)
Supplement: S7 Fig — (A) Single-cell diagram. A pyramidal neuron receives input I and integrates it through a function gdend. (B) Dendritic transformation function gdend as a function of the input I for linear dendrite (left, red) and nonlinear dendrites (right, green). (C) Spatial correlation between laps for blocks of 10 laps on simulations with nonlinear dendrites (green) and linear dendrites (red). Thick lines show averages over 200 cells for each group. Thin lines are individual cells. Note that the spatial correlation for several cells with linear dendrites does not increase over lap blocks. (D) Examples of individual pyramidal cells with linear dendrites. Top, evolution of neuron firing rate over laps as a function of the animal position. Middle, average neuron firing rate over the last 10 laps of exploration as a function of the animal position. Spatial correlation between laps for blocks of 10 laps. (E) Examples of individual pyramidal cells with nonlinear dendrites. Top, evolution of neuron firing rate over laps as a function of the animal position. Middle, average neuron firing rate over the last 10 laps of exploration as a function of the animal position. Spatial correlation between laps for blocks of 10 laps. (PDF) [file pcbi.1007955.s007.pdf]

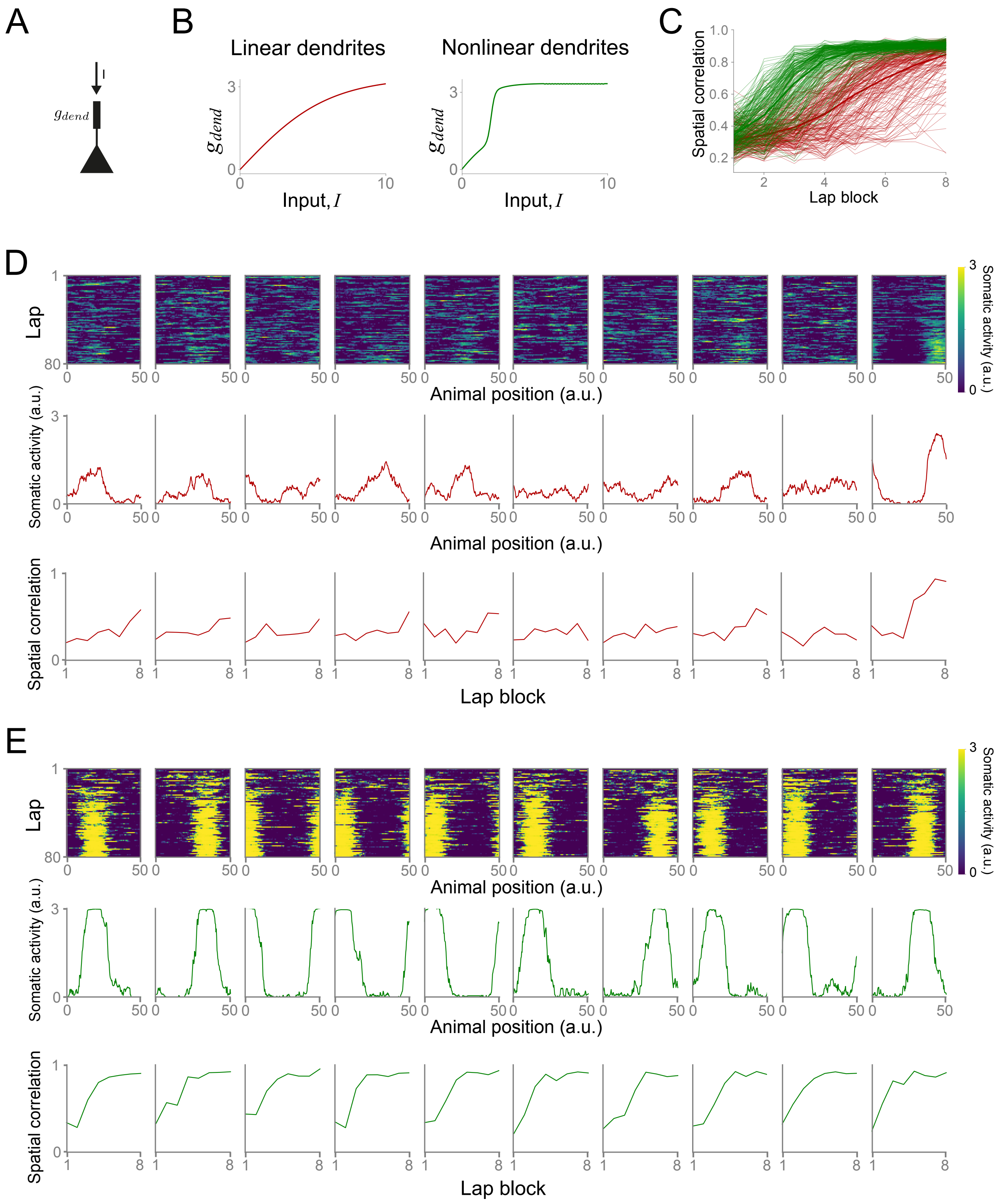
A
I
g_{dend}
B
Linear dendrites
Nonlinear dendrites
g_{dend}
Input, I
C
Spatial correlation
Lap block
D
Lap
Animal position (a.u.)
Somatic activity (a.u.)
Spatial correlation
Lap block
E
Lap
Animal position (a.u.)
Somatic activity (a.u.)
Spatial correlation
Lap block

Figure S7. Dendritic non-linearity leads to reliable place field development. **(A)** Single-cell diagram. A pyramidal neuron receives input I and integrates it through a function g_{dend}. **(B)** Dendritic transformation function g_{dend} as a function of the input I for linear dendrite (left, red) and nonlinear dendrites (right, green). **(C)** Spatial correlation between laps for blocks of 10 laps on simulations with nonlinear dendrites (green) and linear dendrites (red). Thick lines show averages over 200 cells for each group. Thin lines are individual cells. Note that the spatial correlation for several cells with linear dendrites does not increase over lap blocks. **(D)** Examples of individual pyramidal cells with linear dendrites. Top, evolution of neuron firing rate over laps as a function of the animal position. Middle, average neuron firing rate over the last 10 laps of exploration as a function of the animal position. Spatial correlation between laps for blocks of 10 laps. **(E)** Examples of individual pyramidal cells with nonlinear dendrites. Top, evolution of neuron firing rate over laps as a function of the animal position. Middle, average neuron firing rate over the last 10 laps of exploration as a function of the animal position. Spatial correlation between laps for blocks of 10 laps.
