# Supplementary material for: The interplay between somatic and dendritic inhibition promotes the emergence and stabilization of place fields
Source: PLoS Comput Biol. 2020 Jul 10;16(7):e1007955. doi: 10.1371/journal.pcbi.1007955 (PMC7386595; doi:10.1371/journal.pcbi.1007955)
Supplement: S8 Fig — (A-D) Evolution of place fields for the case in which an extra current is applied to the postsynaptic neuron while the animal traverses a section of the track. Yellow bar indicates the induction region in which the extra current is applied. Dashed line indicates the position of the peak of the initial place field. Blue arrow indicates the first induction lap. Red curve shows the evolution of the novelty signal over laps. (A) Place field evolution for 10 induction laps and small induction region (15% of the track). Place fields are shifted towards new position determined by the region of extra current application. (B) Place field evolution for 2 induction laps and small induction region (15% of the track). Place fields are transiently removed by the application of extra current and reemerge at the initial location. (C) Place field evolution for 3 induction lap and small induction region (15% of the track). Place fields are removed following the application of extra current. (D) Same as Fig 5I for a larger number of laps. Place field evolution for 5 induction laps and large induction region (45% of the track). The induction protocol is applied on lap 5, while the novelty signal is still strong. Place fields are shifted to new location. (E-F) Evolution of place fields for a simulation in which dendritic inhibition is suppressed while the animal traverses a section of the track. The disinhibition is induced only after the initial place field has been developed and the amplitude of the novelty signal is negligible. Blue bar indicates the induction region in which dendritic inhibition is suppressed. Dashed line indicates the position of the peak of the initial place field. Blue arrow indicates the first induction lap. Red curve shows the evolution of the novelty signal over laps. (F) Place field evolution for 5 induction laps. Place fields are removed following the suppression protocol. (G) Place field evolution for 15 induction laps. Place fields are shifted towards new [file pcbi.1007955.s008.pdf]

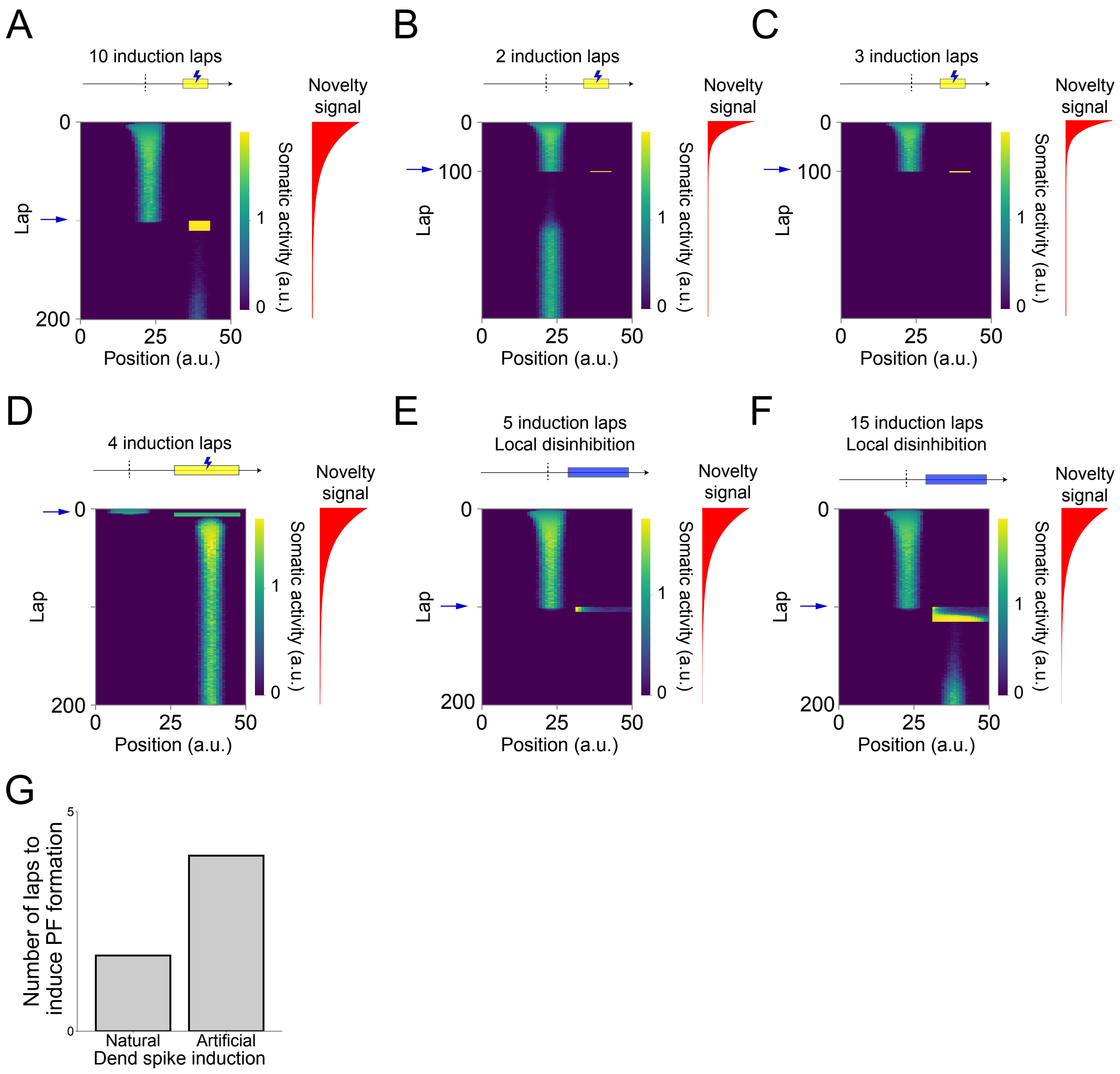

Figure S8 (related to figure 5). Artificially induced CA1 single cell activity can shift place field location. (A-D) Evolution of place fields for the case in which an extra current is applied to the postsynaptic neuron while the animal traverses a section of the track. Yellow bar indicates the induction region in which the extra current is applied. Dashed line indicates the position of the peak of the initial place field. Blue arrow indicates the first induction lap. Red curve shows the evolution of the novelty signal over laps. **(A)** Place field evolution for 10 induction laps and small induction region (15% of the track). Place fields are shifted towards new position determined by the region of extra current application. **(B)** Place field evolution for 2 induction laps and small induction region (15% of the track). Place fields are transiently removed by the application of extra current and reemerge at the initial location. **(C)** Place field evolution for 3 induction lap and small induction region (15% of the track). Place fields are removed following the application of extra current. **(D)** Same as figure 5I for a larger number of laps. Place field evolution for 5 induction laps and large induction region (45% of the track). The induction protocol is applied on lap 5, while the novelty signal is still strong. Place fields are shifted to new location. **(E-F)** Evolution of place fields for a simulation in which dendritic inhibition is suppressed while the animal traverses a section of the track. The disinhibition is induced only after the initial place field has been developed and the amplitude of the novelty signal is negligible. Blue bar indicates the induction region in which dendritic inhibition is suppressed. Dashed line indicates the position of the peak of the initial place field. Blue arrow indicates the first induction lap. Red curve shows the evolution of the novelty signal over laps. **(F)** Place field evolution for 5 induction laps. Place fields are removed following the suppression protocol. **(G)** Place field evolution for 15 induction laps. Place fields are shifted towards new position.
