# Supplementary material for: The interplay between somatic and dendritic inhibition promotes the emergence and stabilization of place fields
Source: PLoS Comput Biol. 2020 Jul 10;16(7):e1007955. doi: 10.1371/journal.pcbi.1007955 (PMC7386595; doi:10.1371/journal.pcbi.1007955)
Supplement: S9 Fig — (A) Single-cell diagram. A pyramidal neuron receives input I and integrates it through a function gdend. (B) Diagram of gdend as a function of the input I (see Methods). α1 controls the linear gain of the dendritic compartment; α2 controls the amplitude of the non-linear term related to dendritic spikes; and I0 controls the minimum input to elicit dendritic spikes. (C) Place field stability analysis. For each measurement of place field stability (see Methods) we perform the following steps: (i) we simulate one lap of exploration, without plasticity; (ii) we measure the place field of the postsynaptic neuron; (iii) we rescale this place field such that its peak is set to 1; (iv) we change the state of the network by adding noise to it; (v-vi) we repeat (ii)-(iii); (vii) we calculate the absolute distance between the two rescaled receptive fields. (PDF) [file pcbi.1007955.s009.pdf]

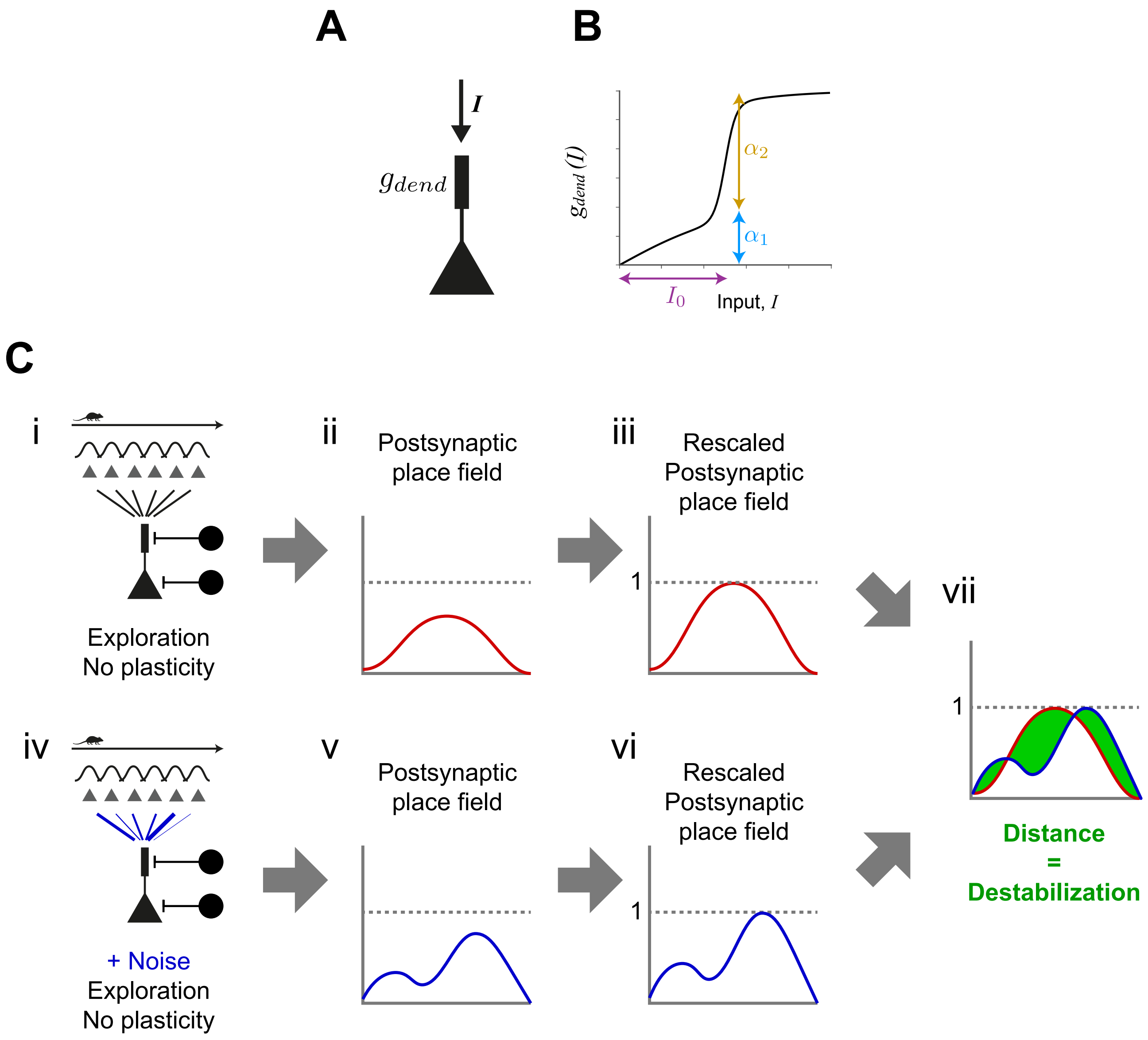

Figure S9. Dendritic non-linearity and stability analysis procedure. **(A)** Single-cell diagram. A pyramidal neuron receives input I and integrates it through a function g_{dend}. **(B)** Diagram of g_{dend} as a function of the input I (see methods). α_1 controls the linear gain of the dendritic compartment; α_2 controls the amplitude of the non-linear term related to dendritic spikes; and I_0 controls the minimum input to elicit dendritic spikes. **(C)** Place field stability analysis. For each measurement of place field stability (see methods) we perform the following steps: (i) we simulate one lap of exploration, without plasticity; (ii) we measure the place field of the postsynaptic neuron; (iii) we rescale this place field such that its peak is set to 1; (iv) we change the state of the network by adding noise to it; (v-vi) we repeat (ii)-(iii); (vii) we calculate the absolute distance between the two rescaled receptive fields.
