# Supplementary material for: The interplay between somatic and dendritic inhibition promotes the emergence and stabilization of place fields
Source: PLoS Comput Biol. 2020 Jul 10;16(7):e1007955. doi: 10.1371/journal.pcbi.1007955 (PMC7386595; doi:10.1371/journal.pcbi.1007955)
Supplement: S1 Methods — (PDF) [file pcbi.1007955.s010.pdf]

Supplementary methods S1

Biophysical neuron model

In our simulations of biophysical neurons, we used the Brian2 library [Goodman 2008] in Python. All the simulations were performed in python and the code will be made available on ModelDB.

Neuron model and morphology

We simulated a ball-and-stick neuron composed of a somatic spherical compartment of diameter 12 μm and an extended dendritic cylinder of diameter 1 μm and length 600 μm modelled using 100 segments. The dendritic cylinder was subdivided in two parts with equal length and each part was set with a specific channel distribution. This was to simulate proximal and apical dendrites. However, the results found in our simulations are not dependent on this subdivision. In our simulations, we included voltage-gated sodium and potassium channels. The passive parameters and the distribution of ion channels used in our simulations can be found in table S1.

Synaptic inputs

The CA1 neuron received inputs from N_{pre} input neurons and these synaptic inputs were modelled as continuous currents being injected at the first dendritic segment (far from the soma). The currents from input neurons were spatially-tuned and each input neuron was preferentially tuned to one position such that the activity of all input neurons together would span over the entire circular track. The tuning of all input currents have the same width, σ_{pre}, and amplitude, A_{pre}. We assume that the animal explores an annular track of length L with speed v. The current delivered by an input neuron i with place field centered at p_0 is

$$I_i^{input}(p) = w_i A_{pre} \exp\left(-\frac{d^2}{2\sigma_{pre}^2}\right) + \xi \ ,$$

where w_i is the respective synaptic weight, p is the animal's position, d is the distance, along the track, between the animal's position and the center of the place field, and ξ is a noise term modelled as an Ornstein-Uhlenbeck processes with mean zero, time constant 100 ms and variance 0.5.

Besides the excitatory input the simulated neurons received dendritic and somatic inhibition. The dendritic inhibition was delivered onto the first dendritic segment, whereas the somatic inhibition was delivered directly at the somatic compartment. Both dendritic and somatic inhibition evolved across time following a novelty signal as defined in our rate-based model. Dendritic inhibition was initially weak and gradually increased, whereas somatic inhibition was initially strong and gradually decreased.

The values for the input place fields and the initial and target dendritic and somatic inhibition can be found in table S1.

Plasticity model

In our simulations of biophysical neurons, we considered an excitatory plasticity rule dependent on coincident pre- and postsynaptic activity. To implement this plasticity rule, synaptic weights were updated at every postsynaptic spike such that

$$\Delta w_i = \eta_{ex} I_i^{input} - \eta_{homeo}\left(\sum_i w_i - \theta_{homeo}\right)/N_{pre} \qquad ,$$

where η_{ex} is the excitatory plasticity learning rate, I_i^{input} is the amplitude of the current from input neuron i, η_{homeo} is the homeostatic plasticity learning rate, and θ_{homeo} is a homeostatic target value for the sum of incoming synaptic weights. All synaptic weights were also bounded between zero and 100.

The values for the parameters for the plasticity model can be found in table S1.

Network with novelty signal at input neurons

The effect of the novelty signal over inhibitory neurons leads to a rapid increase of pyramidal cell activity and a slower return to baseline level. To simulate the effect of a novelty signal spread across multiple brain regions, we included one extra current acting onto excitatory input neurons. This current is considered to be initially stronger and slowly decay to zero following the same time course as the novelty signal acting on CA1 interneurons.

Recurrent network (feedback inhibition)

We explored the effect of feedback inhibition in our simulations by introducing synaptic connections between CA1 pyramidal cells and dendrite-targeting interneurons (thought of as representing somatostatin-expressing cells). To implement this recurrent connections, we assumed that the amplitude of the dendritic inhibition was given by the sum of a novelty-driven term and pyramidal cell-activity-dependent term

$$I_{dend} = I_{dend}^{novelty} + w^{IE} r_{soma}^{pyr} \qquad ,$$

where $I_{dend}^{novelty}$ is the dendritic inhibition described in the methods, w^{IE} is the synaptic weight between the CA1 pyramidal cell and the dendrite-targeting inhibitory neuron, and r_{soma}^{pyr} is the somatic activity of the CA1 pyramidal neuron.

Behavioral-time-scale plasticity (BTSP)

A novel form of synaptic plasticity has been recently shown to span over seconds [1]. To consider the effect of synaptic with such a long time scale, we implemented a plasticity rule that depends on the past activity of both the presynaptic neuron and the postsynaptic dendritic compartment. In this model, the synaptic weight from input neuron j and postsynaptic neuron i is given by

$$\frac{dw_{ij}}{dt}(t) = \eta_{BTSP} \int_0^\infty r_{dend}^i(t-s) r_j(t) \mathrm{e}^{-s/\tau_{BTSP}} ds + \eta_{BTSP} \int_0^\infty r_{dend}^i(t) r_j(t-s) \mathrm{e}^{-s/\tau_{BTSP}} ds - \eta_{homeo} \left(\sum_j w_{ij} - \theta_{homeo} \right) ,$$

where $\eta_{BTSP} = 2 \times 10^{-4}$ ms^{-1} is the BTSP learning rate, τ_{BTSP} is the BTSP time constant, η_{homeo} is the learning rate of the homeostatic term, and θ_{homeo} is a target homeostatic constant.

In our simulations, we considered two cases: $\tau_{BTSP} = 1.5$s and $\tau_{BTSP} = 1.0$s. The remaining parameters were kept at the same values as the ones used for the main simulations described in the methods.

Natural versus artificial dendritic spikes

Artificial dendritic spike were induced by injecting an extra, depolarizing current at the dendritic compartment. Natural dendritic spikes were detected when the dendritic activity crossed a threshold value defined at 1.0.

References

1. Bittner K, Milstein A, Grienberger C, Romani S, Magee J. Behavioral time scale synaptic plasticity underlies CA1 place fields. Science. 2017;357(6355):1033-1036.
