# Supplementary material for: The interplay between somatic and dendritic inhibition promotes the emergence and stabilization of place fields
Source: PLoS Comput Biol. 2020 Jul 10;16(7):e1007955. doi: 10.1371/journal.pcbi.1007955 (PMC7386595; doi:10.1371/journal.pcbi.1007955)
Supplement: S1 Table — (PDF) [file pcbi.1007955.s011.pdf]

Table S1: Parameters for biophysical, spatially extended neuron model implemented in Python using Brian2 [1].

Neuron Model		
**Name**	**Value**	**Description**
dt	0.1 ms	Time step
g_L	0.3 mS cm^{-2}	Leak conductance
E_L	- 65 mV	Leak potential
C_m	1.28 μF cm^{-2}	Membrane capacitance
R_i	100 Ω	Intracellular resistivity
**Ion channels**		
**Name**	**Value**	**Description**
E_{Na}	55 mV	Reversal potential for Sodium channels
E_K	- 77 mV	Reversal potential for Potassium channels
g_{Na} (soma)	40 mS cm^{-2}	Maximum sodium conductance (soma)
g_K (soma)	35 mS cm^{-2}	Maximum potassium conductance (soma)
g_{Na} (proximal dend)	30 mS cm^{-2}	Maximum sodium conductance for the proximal half of the dendrite
g_K (proximal dend)	25 mS cm^{-2}	Maximum potassium conductance for the proximal half of the dendrite
g_{Na} (distal dend)	52 mS cm^{-2}	Maximum sodium conductance for the distal half of the dendrite
g_K (distal dend)	35 mS cm^{-2}	Maximum potassium conductance for the distal half of the dendrite
**Plasticity Model**		
**Name**	**Value**	**Description**
η_{ex}	0.18	Excitatory plasticity learning rate
η_{homeo}	0.015	Homeostatic plasticity learning rate
θ_{homeo}	20.0	Homeostatic target value
**Place-tuned input**		
**Name**	**Value**	**Description**
A_{pre}	2.2	Presynaptic place field amplitude
σ_{pre}	5.0	Presynaptic place field width
**Novelty signal**		
**Name**	**Value**	**Description**
τ_n	100 s	Time constant for novelty signal decay
I^0_{dend}	0.0 A m^{-2}	Initial dendritic inhibition
I^∞_{dend}	2.5 A m^{-2}	Target dendritic inhibition
I^0_{soma}	0.01 A m^{-2}	Initial somatic inhibition
I^∞_{soma}	0.0 A m^{-2}	Target somatic inhibition
**Simulation parameters**		
**Name**	**Value**	**Description**
N_{pre}	10	Number of presynaptic neurons
T_{length}	50 a.u.	Track length (arbitrary units)
v	0.5×10^{-2} ms^{-1}	Animal speed

References

1. Goodman D. Brian: a simulator for spiking neural networks in Python. Frontiers in Neuroinformatics. 2008;2.
