# Supplementary material for: The interplay between somatic and dendritic inhibition promotes the emergence and stabilization of place fields
Source: PLoS Comput Biol. 2020 Jul 10;16(7):e1007955. doi: 10.1371/journal.pcbi.1007955 (PMC7386595; doi:10.1371/journal.pcbi.1007955)
Supplement: Attachment — Submitted filename: Response_to_revewiers.pdf [file pcbi.1007955.s012.pdf]

General comments

We are again grateful for the reviewers' comments and suggestions. Below, we provide a point-by-point response to all comments, suggestions and concerns raised by the reviewers. Our response has been written in blue font. All references to figures and line numbers refer to the revised version of the manuscript.

Reviewer #1:

- as the authors' reply has confirmed, in this single cell model, the place field is simply inherited from the place field of one of the presynaptic neurons. Hence the model does not describe the mechanism underlying the formation of place fields, it assumes place fields are already present in other cells. For me this is a major flaw with respect to the claims made throughout the paper.

Even though CA1 pyramidal cells mainly inherit upstream place fields in our model, these neurons perform nonlinear computations on 3 stages: dendritic nonlinearities, nonlinear dendrite-to-soma propagation, and somatic rectification. Furthermore, CA1 pyramidal cells do not select exclusively one connection from input neurons. Instead, it combines these inputs. We appreciate, however, that these cells might receive more complex inputs, and, in the revised version of the manuscript, we add the paragraph below in which we clearly state the current limitation of our model and discuss possible extensions and their consequences (l496-506).

"Following synaptic plasticity, CA1 pyramidal cells might exhibit spatially-tuned activity in our model. The place fields displayed by these neurons is the result of a combination of place fields exhibited by upstream neurons. This combination is then processed by the CA1 neuron in three stages: dendritic nonlinearities, dendrite-to-soma nonlinear propagation, and somatic rectification. These nonlinearities form important processing steps and ultimately lead to the formation of silent and place cells. To simplify our results and analysis, we impose single-peaked, spatially-tuned activity on input neurons. CA1 pyramidal cells, however, are also subject to more complex inputs such as grid-like inputs from entorhinal cortex or from multi-peaked CA3 place cells. The incorporation of these complex inputs in our model would likely lead to more complex CA1 pyramidal cell place fields. In this case, both dendrites and soma could exhibit multi-peaked place-tuned activity, as observed experimentally (Rashid et al., 2020). Future extensions of our model could explore the effect of multi-peaked place-tuned inputs on place field plasticity and remapping."

- a large number of ad-hoc ingredients is added to capture details of experimental results. As the authors have clarified in the reply, each ingredient was added to capture some of the experimental results, hence it is not surprising that the model captures many experimental results.

While conceptualizing our model, we aimed to impose as little assumptions as possible while still capturing several experimental results. Each ingredient in our model is a phenomenological abstraction of a more complex phenomenon. Therefore, we believe that our model is capable of reproducing a wide range of experimental data with the advantage of being simple. Moreover, albeit the simplicity, our model provides testable predictions.

- the posited mechanisms are not testable experimentally as they are based on quantities that cannot be

accessed in vivo (plasticity, inputs from different interneuron classes, activity in the dendritic compartment).

We agree that some of the elements of the model might be challenging to be tested experimentally. The quantities cited, however, could be probed either indirectly or even directly. Place-tuned activity in dendrites, for example, have been recently measured using calcium imaging (Rashid et al., 2020) whereas inputs from different interneuron classes were measured in CA1 pyramidal cells *in vitro* (Udakis et al., 2019).

- testable experimental predictions are very limited

In the revised version of the manuscript, we included an extra paragraph listing the main prediction from our model and suggestions of experiments to test those predictions (l425-441). Extra text included:

"Our model provides several predictions that could be tested experimentally. In our simulations, place fields can be formed even in the absence of a novelty signal (figure S1A). However, the dynamics of the evolution of place fields are altered in this scenario. For instance, the amplitude of newly developed place fields does not progress towards a lower level with spatial exploration (figure S1A). Although controlling the novelty signal experimentally --- by modulating both SST and PV interneuron activity simultaneously --- would be extremely challenging, this prediction could be tested by analyzing place fields formed after several laps of exploration, when the novelty signal could be neglected and the interneuron activity would have returned to its baseline level \cite{Sheffield2017}. Furthermore, in our simulations, the quick and abrupt switch from silent to place cell is a direct consequence of the nonlinear propagation of inputs from dendrites to soma. This non-linearity has been shown to be mediated by a persistent sodium current (Hsu et al., 2018). In the absence of this gating mechanism, place field amplitude would vary across a wider range of amplitudes (figure S1B). This could be tested by measuring place field amplitudes during the suppression of the persistent sodium current in single cells while the animal explored an environment (Hsu et al., 2018). Finally, our model provides specific prediction on the efficiency of perturbation protocols to shift place field location. Although place fields have been manipulated experimentally by single cell stimulation (Diamantaki et al., 2018), our model provides a framework that can be used to designing further protocols."

- alternative models are listed, but it is not explained how the different models can be distinguished experimentally

In our discussion, we argue that our model does not contradict the previous models presented. Instead, those models could act in complementary ways. Therefore, future experiments would not necessarily distinguish those models but rather exclude or support parts of a wider model formed by the combination of those models.

Reviewer #2:

l.173, "Given that the amplitude of the injected current is above a certain threshold, [...]": The authors probably mean to say "If / Under the condition that the amplitude..."

We have taken the suggestion of the reviewer and modified the text to “Under the condition that …”.

Reviewer #3:

While the paper is now definitely better, the new insgihts remain incremental and will have to await for experimental validation.

We appreciate the reviewer’s comment and we agree that it would be important to seek experimental validation for the predictions proposed by our model. We believe, however, that our model brings new insights into the mechanisms underlying place field development and that we propose several testable prediction that could be taken further by experimental groups.
