# Supplementary material for: The interplay between somatic and dendritic inhibition promotes the emergence and stabilization of place fields
Source: PLoS Comput Biol. 2020 Jul 10;16(7):e1007955. doi: 10.1371/journal.pcbi.1007955 (PMC7386595; doi:10.1371/journal.pcbi.1007955)
Supplement: Attachment — Submitted filename: Response_to_reviewers.pdf [file pcbi.1007955.s013.pdf]

General comments

We are grateful for the reviewer's comments and suggestions. Below, we provide a point-by-point response to all comments and suggestions raised by the reviewer. Our response has been written in blue font. All references to figures and line numbers refer to the revised version of the manuscript.

Reviewer #1:

Abstract: "We show that although somatic disinhibition is sufficient to form place cells"

Introduction: "We show that somatic disinhibition is sufficient to form place cells"

I think "place fields" would be clearer than "place cells" here. Also, the authors should add some text to indicate that somatic disinhibition is sufficient to form place fields from / assuming / along with spatially modulated inputs

We agree with the reviewer and we have replaced the term place cells with place fields (l.15-16 and l.118). We have also added one extra sentence to indicate that our model requires spatially tuned inputs (l.118).

Introduction: "If a spatially uniform current is applied to a silent cell, however, this cell starts to produce place-tuned activity [Lee et al., 2012]. This transition from silent to place cell is abrupt and once the silent cell is turned into a place cell, the amplitude of the place field is fairly independent of the amplitude of the applied current [Lee et al., 2012]"

I think it would improve the clarity of the manuscript if the authors also mentioned here that, once the spatially uniform current is removed, the place field disappears

We fully agree with the reviewer and we have included one extra sentence to improve the clarity of the manuscript (l.52-53).

Introduction: "All these models, however, do not take into account individual interneuron types and their modulation during exploration of novel environments"

This statement may be true, but it is also misleading, as several of these models do account for the origin of spatially modulated inputs to place cells, which are assumed here. Hence, I would say that those models complement (and are compatible with) the model presented here, rather than being superseded by it. The authors should add some text to clarify this point.

To make clear the contributions of previous models. To clearly state that our model is not conflicting with previous models, but instead complementing them, we added one extra sentence in the introduction (l.112-113) and one extra sentence in the discussion (l.491-492).

Introduction: "subthreshold hill of depolarization"

I think "subthreshold ramp of depolarization" would be clearer / more consistent with existing literature

The sentence has been modified in the revised version of the manuscript (l.90).

Discussion: "The place fields displayed by these neurons ARE the result of a combination of place fields exhibited by upstream neurons"

This has now been rectified (l.499).
