# Supplementary material for: The interplay between somatic and dendritic inhibition promotes the emergence and stabilization of place fields
Source: PLoS Comput Biol. 2020 Jul 10;16(7):e1007955. doi: 10.1371/journal.pcbi.1007955 (PMC7386595; doi:10.1371/journal.pcbi.1007955)
Supplement: Peer review history

# Author comment: Author response to previous submission

**DOI:** 10.1371/journal.pcbi.1007955.r001

21 Apr 2020

Attachment Submitted filename: Response_to_revewiers.pdf Click here for additional data file.

# Editor report: Decision Letter 0

**DOI:** 10.1371/journal.pcbi.1007955.r002

**Authors:** Kim T. Blackwell, Daniel Bush

4 May 2020

Dear Dr. Clopath,

Thank you very much for submitting your manuscript "The interplay between somatic and dendritic inhibition promotes the emergence and stabilization of place fields" for consideration at PLOS Computational Biology. Your manuscript was reviewed by members of the editorial board, who appreciated the attention to an important topic. Based on the reviews, we are likely to accept this manuscript for publication, providing that you modify the manuscript according to the review recommendations. Please see below for those final minor comments. 

Please prepare and submit your revised manuscript within 30 days. If you anticipate any delay, please let us know the expected resubmission date by replying to this email. 

When you are ready to resubmit, please upload the following:

[1] A letter containing a detailed list of your responses to all review comments, and a description of the changes you have made in the manuscript. Please note while forming your response, if your article is accepted, you may have the opportunity to make the peer review history publicly available. The record will include editor decision letters (with reviews) and your responses to reviewer comments. If eligible, we will contact you to opt in or out

[2] Two versions of the revised manuscript: one with either highlights or tracked changes denoting where the text has been changed; the other a clean version (uploaded as the manuscript file).

Important additional instructions are given below your reviewer comments.

Thank you again for your submission to our journal. We hope that our editorial process has been constructive so far, and we welcome your feedback at any time. Please don't hesitate to contact us if you have any questions or comments.

Sincerely,

Daniel Bush

Associate Editor

PLOS Computational Biology

Kim Blackwell

Deputy Editor

PLOS Computational Biology

***********************

A link appears below if there are any accompanying review attachments. If you believe any reviews to be missing, please contact ploscompbiol@plos.org immediately:

[LINK]

Abstract: "We show that although somatic disinhibition is sufficient to form place cells"

Introduction: "We show that somatic disinhibition is sufficient to form place cells"

I think "place fields" would be clearer than "place cells" here. Also, the authors should add some text to indicate that somatic disinhibition is sufficient to form place fields from / assuming / along with spatially modulated inputs

Introduction: "If a spatially uniform current is applied to a silent cell, however, this cell starts to produce place-tuned activity [Lee et al., 2012]. This transition from silent to place cell is abrupt and once the silent cell is turned into a place cell, the amplitude of the place field is fairly independent of the amplitude of the applied current [Lee et al., 2012]"

I think it would improve the clarity of the manuscript if the authors also mentioned here that, once the spatially uniform current is removed, the place field disappears

Introduction: "All these models, however, do not take into account individual interneuron types and their modulation during exploration of novel environments"

This statement may be true, but it is also misleading, as several of these models do account for the origin of spatially modulated inputs to place cells, which are assumed here. Hence, I would say that those models complement (and are compatible with) the model presented here, rather than being superseded by it. The authors should add some text to clarify this point.

Introduction: "subthreshold hill of depolarization"

I think "subthreshold ramp of depolarization" would be clearer / more consistent with existing literature

Discussion: "The place fields displayed by these neurons ARE the result of a combination of place fields exhibited by upstream neurons"

Figure Files:

While revising your submission, please upload your figure files to the Preflight Analysis and Conversion Engine (PACE) digital diagnostic tool, https://pacev2.apexcovantage.com. PACE helps ensure that figures meet PLOS requirements. To use PACE, you must first register as a user. Then, login and navigate to the UPLOAD tab, where you will find detailed instructions on how to use the tool. If you encounter any issues or have any questions when using PACE, please email us at figures@plos.org.

Data Requirements:

Please note that, as a condition of publication, PLOS' data policy requires that you make available all data used to draw the conclusions outlined in your manuscript. Data must be deposited in an appropriate repository, included within the body of the manuscript, or uploaded as supporting information. This includes all numerical values that were used to generate graphs, histograms etc.. For an example in PLOS Biology see here: http://www.plosbiology.org/article/info%3Adoi%2F10.1371%2Fjournal.pbio.1001908#s5.

Reproducibility:

To enhance the reproducibility of your results, PLOS recommends that you deposit laboratory protocols in protocols.io, where a protocol can be assigned its own identifier (DOI) such that it can be cited independently in the future. For instructions see http://journals.plos.org/ploscompbiol/s/submission-guidelines#loc-materials-and-methods

# Author comment: Author response to Decision Letter 0

**DOI:** 10.1371/journal.pcbi.1007955.r003

14 May 2020

Attachment Submitted filename: Response_to_reviewers.pdf Click here for additional data file.

# Editor report: Decision Letter 1

**DOI:** 10.1371/journal.pcbi.1007955.r004

**Authors:** Kim T. Blackwell, Daniel Bush

15 May 2020

Dear Dr. Clopath,

We are pleased to inform you that your manuscript 'The interplay between somatic and dendritic inhibition promotes the emergence and stabilization of place fields' has been provisionally accepted for publication in PLOS Computational Biology.

Before your manuscript can be formally accepted you will need to complete some formatting changes, which you will receive in a follow up email. A member of our team will be in touch with a set of requests.

Please note that your manuscript will not be scheduled for publication until you have made the required changes, so a swift response is appreciated.

IMPORTANT: The editorial review process is now complete. PLOS will only permit corrections to spelling, formatting or significant scientific errors from this point onwards. Requests for major changes, or any which affect the scientific understanding of your work, will cause delays to the publication date of your manuscript.

Should you, your institution's press office or the journal office choose to press release your paper, you will automatically be opted out of early publication. We ask that you notify us now if you or your institution is planning to press release the article. All press must be co-ordinated with PLOS.

Thank you again for supporting Open Access publishing; we are looking forward to publishing your work in PLOS Computational Biology. 

Best regards,

Daniel Bush

Associate Editor

PLOS Computational Biology

Kim Blackwell

Deputy Editor

PLOS Computational Biology

***********************************************************

# Editor report: Acceptance letter

**DOI:** 10.1371/journal.pcbi.1007955.r005

**Authors:** Kim T. Blackwell, Daniel Bush

29 Jun 2020

PCOMPBIOL-D-20-00642R1 

The interplay between somatic and dendritic inhibition promotes the emergence and stabilization of place fields

Dear Dr Clopath,

I am pleased to inform you that your manuscript has been formally accepted for publication in PLOS Computational Biology. Your manuscript is now with our production department and you will be notified of the publication date in due course.

The corresponding author will soon be receiving a typeset proof for review, to ensure errors have not been introduced during production. Please review the PDF proof of your manuscript carefully, as this is the last chance to correct any errors. Please note that major changes, or those which affect the scientific understanding of the work, will likely cause delays to the publication date of your manuscript. 

Soon after your final files are uploaded, unless you have opted out, the early version of your manuscript will be published online. The date of the early version will be your article's publication date. The final article will be published to the same URL, and all versions of the paper will be accessible to readers.

Thank you again for supporting PLOS Computational Biology and open-access publishing. We are looking forward to publishing your work! 

With kind regards,

Laura Mallard

PLOS Computational Biology | Carlyle House, Carlyle Road, Cambridge CB4 3DN | United Kingdom ploscompbiol@plos.org | Phone +44 (0) 1223-442824 | ploscompbiol.org | @PLOSCompBiol
